# Planning dam portfolios for low sediment trapping shows limits for sustainable hydropower in the Mekong

**DOI:** 10.1126/sciadv.aaw2175

**Published:** 2019-10-23

**Authors:** R. J. P. Schmitt, S. Bizzi, A. Castelletti, J. J. Opperman, G. M. Kondolf

**Affiliations:** 1 Natural Capital Project, Department of Biology and the Woods Institute for the Environment, Stanford University, Stanford, CA, USA.; 2 Department of Landscape Architecture and Environmental Planning, University of California, Berkeley, Berkeley, CA, USA.; 3 Department of Electronics, Information, and Bioengineering, Politecnico di Milano, Milano, Italy.; 4 Department of Geosciences, University of Padova, Padua, Italy.; 5 Institute of Environmental Engineering, ETH Zurich, Zurich, Switzerland.; 6 WWF Global Science, Chagrin Falls, Ohio, USA.; 7 Collegium–Lyon Institute for Advanced Study, University of Lyon, Lyon, France.

## Abstract

The transboundary Mekong Basin has been dubbed the “Battery of Southeast Asia” for its large hydropower potential. Development of hydropower dams in the six riparian countries proceeds without strategic analyses of dam impacts, e.g., reduced sediment delivery to the lower Mekong. This will impact some of the world’s largest freshwater fisheries and endangers the resilience of the delta, which supports 17 million livelihoods, against rising sea levels. To highlight alternatives, we contribute an optimization-based framework for strategic sequencing of dam development. We quantify lost opportunities from past development and identify remaining opportunities for better tradeoffs between sediment and hydropower. We find that limited opportunities remain for less impactful hydropower in the lower basin, where most development is currently planned, while better trade-offs could be reached with dams in the upper Mekong in China. Our results offer a strategic vision for hydropower in the Mekong, introduce a globally applicable framework to optimize dam sequences in space and time, and highlight the importance of strategic planning on multiple scales to minimize hydropower impacts on rivers.

## Teaser

Strategic planning in the Mekong Basin could improve trade-offs between hydropower and sediment supply to the Mekong Delta.

## INTRODUCTION

The Mekong River supports the largest freshwater fishery on earth and is second in fish species richness only to the much larger Amazon River Basin (*1*). The river transports a high load of sediment and associated nutrients, which support the productive ecosystems and fisheries of the lower Mekong floodplains and the Tonle Sap Lake (*2*). Sediment deposition in the Mekong Delta is also essential for offsetting the effects of land subsidence and sea level rise (*3*). The Mekong Basin also has large potential for hydropower generation [268,000 gigawatt hours of electricity per year (GWh/year)], of which around half has been developed (*4*, *5*). Existing dams have already reduced the river’s sediment load, and recent measurements show declining sediment supply to the Mekong Delta (*6*) and a rapidly receding delta coastline (*7*). A full build-out of Mekong hydropower would result in an even greater (>90%) reduction of sediment transport to the delta (*8*). Much of that reduction would be associated with the construction of very large dams on the mainstem of the lower Mekong, several of which are planned for the near future.

Such a reduction would further increase the risk that most of the delta could subside below sea level by the end of the century (*3*). This subsidence carries major socioeconomic risks, given that the delta supports a population of at least 17 million people, a substantial industrial production, and more than 50% of Vietnam’s rice harvest (2.5% of global production) (*2*). Such a reduction in sediment transport would also have major impacts on fisheries in Tonle Sap Lake and the lower Mekong floodplains (*9*). Hence, there are substantial trade-offs between expanding hydropower generation in the basin and maintaining sediment-related ecosystem services.

Hydropower development in the basin has proceeded essentially project-by-project, without a strategic analysis of cumulative dam impacts and benefits. A strategic environmental assessment of mainstem dams was completed (*10*) but has had limited influence on decision-making. Certainly, this project-by-project development has been partially the result of complex political and socioeconomic realities. The Mekong is a large transboundary basin shared between six countries (China, Laos, Thailand, Myanmar, Cambodia, and Vietnam). In the past, riparian countries focused on exploiting their share of the basin’s hydropower potential, both for meeting domestic energy demand and for exporting power to neighboring countries (*11*), without considering trade-offs between benefits and cumulative basin-scale impacts of dams.

Besides these realities, there is little successful experience to draw upon in large transboundary basins and few tested tools with which to explore trade-offs to identify opportunities for lower impact hydropower (*12*). While prior studies highlighted the impact of future dams on sediment budgets (*8*, *13*, *14*), hydrology (*15*), and food production (*16*, *17*) in the basin, most studies focused on the lower basin and none analyzed a full range of strategic options. However, it has been demonstrated that strategic planning and operation of dams can improve trade-offs between dam impacts and benefits in sub-basins or subgroups of dams in the lower Mekong (*18*–*20*). Missing still is a strategic analysis of hydropower development on the scale of the whole Mekong Basin.

Strategic hydropower planning is rarely applied on basin scales, both because of political challenges and because of improvements needed in planning tools available. For example, the question of how to time development has generally been omitted from strategic planning approaches, except for examples with few dam sites (<10) (*21*). Previous applications of large-scale strategic planning aimed to identify combinations of dam sites, i.e., dam portfolios, that create Pareto-optimal (PO) trade-offs between dam benefits and impacts (*12*, *18*, *20*). While this is a relevant step, the resulting dam portfolios alone do not yield information on how to sequence the development, i.e., which dam to build and when. Hydropower development in a river basin typically proceeds over several decades and may stop short of full basin development. Thus, guidance on the development sequence can result in better trade-offs for whatever portfolio is lastly developed.

**Fig. 1:**
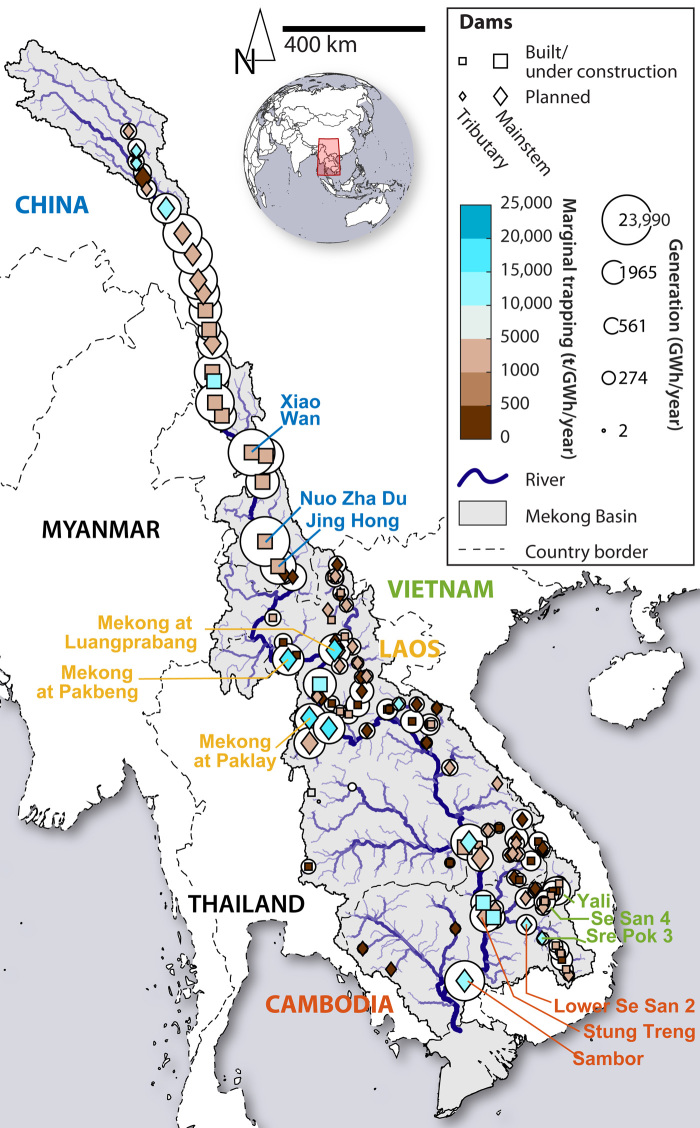
Geography of the Mekong Basin and existing and possible future large dams. The color of dam markers indicates the marginal impact of each dam site in terms of trapped sediment per unit energy generation. Brownish colors indicate more favorable trade-offs, i.e., more sediment passing per generation. The three dams with the largest potential trapping are labeled for each country (country and dam labels share the same color). Countries with black labels do not have any major dam sites. The red rectangle on the globe inset indicates the location of the Mekong Basin.

Building on prior studies demonstrating that system-scale approaches could identify better trade-offs between dam impacts and benefits than project-by-project development (*18*, *20*, *22*), we explored the trade-offs between hydropower generation and dam impacts on sediment transport on the scale of the whole Mekong Basin. We first compiled a database of 124 large dam projects in the basin (Materials and Methods, Fig. 1, figs. S1 and S2, and data S1). Second, we set up the CASCADE (CAtchment Sediment Connectivity And DElivery) sediment routing model for the entire basin (*23*) (Materials and Methods, figs. S3 and S4, and Supplementary Methods 2 and 3). We developed an approach combining CASCADE with a multiobjective evolutionary algorithm (*24*) to derive dam sequences that minimize trade-offs between power generation and sediment supply to the lower Mekong. We present this analysis for different initial conditions, i.e., starting from a pristine basin or a basin with existing dams, and for different scales, i.e., for the entire basin and for the lower basin countries only (Materials and Methods). We then derive optimal dam sequences by post-processing the derived PO dam portfolio (Materials and Methods, Supplementary Method 6, figs. S7 and S8, and Supplementary Result 1). We compared the sharing of benefits from hydropower generation with impacts of dams on sediment transport using a newly introduced index for changing sediment budgets on a whole network scale (Materials and Methods). Last, we discuss opportunities and research challenges for mainstreaming strategic planning worldwide. For example, planners and decision-makers engaged in system-scale planning for energy will be confronted with a range of trade-offs besides sediment trapping, including displacement of communities as well as impacts on migratory fish biomass and biodiversity and terrestrial ecosystems (*1*, *20*). Furthermore, while the present analysis focuses on dam siting, strategic planning can also encompass the optimal design and operation of individual dams (*25*). While this paper focuses on hydropower, planning that considers a broader set of renewable energy options—both in terms of grid performance and trade-offs with other objectives—can help identify options that are low carbon, low cost, and low impact on social and environmental resources (*26*, *27*).

### Political realities and geomorphic heterogeneity in the transboundary Mekong Basin

The great geomorphic and hydrologic diversity and the political realities of the transboundary Mekong Basin are the backdrop for the challenges and opportunities for strategic planning in the basin. The Mekong Basin covers 790,000 km^2^ shared between six countries (Fig. 1). From its sources on the Tibetan Plateau at around 5200 m, the Mekong (called Lancang in China) travels through high-gradient gorges until it reaches the China-Laos border; the portion of the basin downstream of that point is defined as the lower Mekong Basin (LMB). The LMB includes Laos, Thailand, Cambodia, and Vietnam. Myanmar represents a very small part of the LMB. Differences in geology, topographic relief, and climate determine the provenance of sediment delivered to the Mekong Delta and the contribution of each country to the basin’s sediment budget. Sediment data in the basin are scarce, but available evidence suggests that half of the basin’s sediment load originated in the Lancang portion of the basin (*8*), with additional major contributions from the Northern Highlands at the China-Laos border and from tributaries on the lower Mekong that are shared between Laos, Cambodia, and Vietnam (*18*). Last, the lower Mekong floodplains, the Tonle Sap basin in Cambodia, and the Mekong Delta in Vietnam act as sinks for sediment.

Since 1995, the basin countries (except for Myanmar and China) have been members of the Mekong River Commission (MRC). Under the 1995 agreement, the MRC member countries agreed to notify and consult other MRC members about developments with transboundary impacts. However, notifications about dam construction are not comprehensive, member countries often do not adhere to consultation procedures (*28*), and sharing of data, e.g., regarding hydrology and sediment, is incomplete. China holds a powerful position in the basin as the upstream-most country (*15*), and through releases from its major storage reservoirs, China can exert considerable control over the low-flow hydrology of the lower Mekong (*29*). China has observer, not member, status in the MRC and thus participates neither in the consultation process nor in efforts for data and knowledge sharing. The extent to which China will increase transboundary cooperation, such as via new mechanisms such as the Lancang-Mekong Cooperation, remains to be seen (*29*).

## RESULTS

Around 59,000-MW capacity could be installed at the 124 dam sites that we identified, which would result in a generation of around 268,000 GWh/year (Materials and Methods, figs. S1 and S2, Supplementary Method 1, and data S1). Of these possible dams, 32 are currently in operation and 24 are under construction, for a total generation of around 138,000 GWh/year. Most dams operational today are on the lower Lancang and on tributaries in the lower Mekong countries (Fig. 1). The remaining potential sites for large dams are either on the upper lancang (upstream of and in between existing dams) and on the lower Mekong mainstem (Fig. 1). There are 15 undeveloped dam sites in China (around 55,000 GWh/year generation), 40 in Laos (around 53,000 GWh/year generation), and 10 in Cambodia (around 21,000 GWh/year generation). There is one remaining dam site in Vietnam and none in Thailand. Myanmar has no listed existing or potential dams in the basin.

Of all the dams in the basin, those in the Lancang, such as Xiao Wan and Nuo Zha Du, have the greatest potential for trapping sediment due to the high sediment yields from the Tibetan Plateau. However, because of the Lancang’s high gradient and large flow, the electricity generation potential of these dams is also very high. For example, the annual generation of the proposed Nuo Zha Du on the Lancang (23,900 GWh/year) is comparable to all built and potential sites in Cambodia (23,400 GWh/year). The marginal impact in terms of GWh/year of generation per ton of sediment trapped is hence still relatively low for these upstream sites (Fig. 1, brown colors). Dams along the lower Mekong mainstem have much less favorable trade-offs, i.e., they trap much more sediment relative to their power generation (Fig. 1, blue colors). Some lower basin countries are thus limited to a portfolio of dam sites with unfavorable trade-offs between hydropower and sediment trapping.

Quantifying the marginal impact of each dam within a portfolio requires consideration of upstream dams, as these dams control how much sediment will reach a more downstream dam site. Example, Even a dam such as Nuo Zha Du would trap little sediment once other dams with high potential for sediment trapping are built upstream of it. Thus, we simulated the cumulative sediment trapping and hydropower generation along the trajectory of past and ongoing dam developments, i.e., the actual sequence of dam construction that has resulted in the current dam portfolio in the basin (Materials and Methods, fig. S5).

**Fig. 2:**
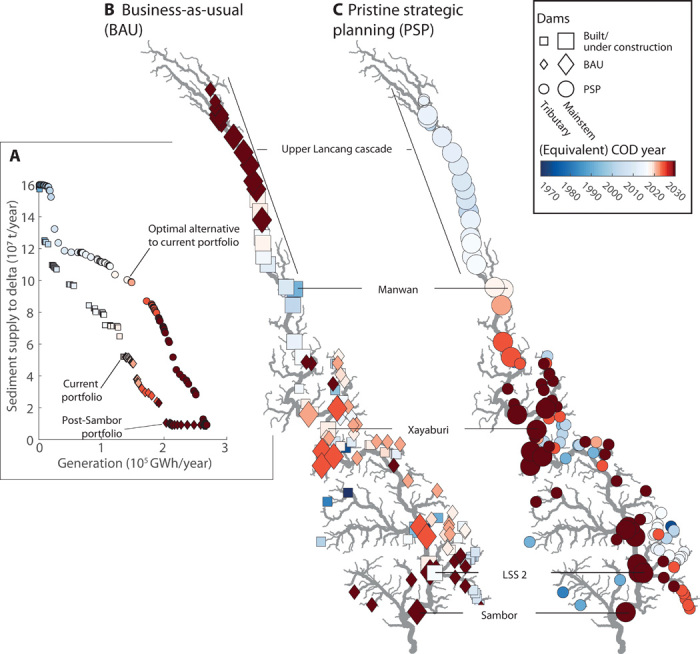
Lost opportunities from nonstrategic hydropower development in the Mekong. (**A**) Trade-offs between hydropower generation and cumulative sediment trapping for different dam sequences. The BAU sequence replicates the past (square markers) and planned future development of dams (diamond markers). The BAU is compared to an optimal sequence starting from a pristine basin, termed PSP (circle markers). The color of each marker indicates the COD year for the BAU, respectively, and the equivalent COD for the PSP sequence (blue hues, past; red hues, future; whitish hues, near past and future). (**B**) Spatiotemporal sequence of dam development for the BAU sequence. (**C**) Optimal spatiotemporal sequence of dam development for the PSP sequence. The color of a dam site in (B) and (C) indicates the COD and hence the sequence of dams [colors are equivalent between (A), (B), and (C)]. LSS 2, Lower Se San 2.

The current dam portfolio will cumulatively reduce sediment supply to the delta from an estimated pre-dam load of 160 to 52 metric megatons (Mt)/year and will generate around 125,000 GWh/year after all projects under construction are completed (Fig. 2A, square markers). The current dam portfolio includes major dams on the lower Lancang and many dams on tributaries to the Lower Mekong River. Near-future dam construction for the business-as-usual (“BAU”) case (Fig. 2A, diamond markers) is focused on mainstem dams on the lower Mekong, which, by around 2025, would reduce the sediment supply to the delta to around 29 Mt/year. Construction of very large dams on the lower Mekong, such as Sambor (Fig. 1), will further reduce the sediment output to 13 Mt/year (Fig. 2A). Construction of additional dams in the upper Lancang cascade in China (Fig. 2B and diamond markers upstream of Xiao Wan Dam in Fig. 1D) is planned for a more distant future. Constructing all dams would trap 95% of the transported sediment, reducing the sediment supply to the delta to around 9 Mt/year. Dams built post-Sambor would have relatively small incremental impact on sediment supply to the delta because Sambor dams would largely control sediment passage to the delta.

### Foregone strategic alternatives to the current hydropower portfolio

To date, dams in the Mekong have been developed project-by-project and hence without strategic planning to account for trade-offs between hydropower generation and sediment trapping or other ecosystem services. This project-by-project development is very likely to result in a dam portfolio with suboptimal trade-offs between hydropower generation and sediment transport (*18*). To study alternatives, we use a novel approach to derive sequences of dam development that maximize sediment supply to the delta for any level of hydropower generation or conversely maximize hydropower generation for any level of sediment supply (Materials and Methods).

Following an optimal development sequence results in nonregret dam portfolios: If further hydropower expansion is not needed (e.g., because of less than projected growth in demand or lower prices of other renewables), development can be stopped at an intermediate portfolio with the lowest possible impact on sediment supply to the delta for that level of generation. This information on optimal sequencing of development was not available from previous approaches (*18*, *20*) (for a detailed explanation of the concept and the dam sequencing method, refer to Materials and Methods, Supplementary Method 5, and figs. S6 and S7). We also compare the commercial operation dates (CODs) along the past and planned dam sequence with the alternative optimal sequences. Hence, we can assign an equivalent COD to any dam added to an alternative sequence (Materials and Methods). This step is not required for the optimization but allows us to estimate when a specific dam in an alternative sequence would need to be developed to match the generation along the planned sequence.

We identify an optimal sequence starting from a pristine basin without any dam development [“Pristine strategic planning (PSP)”] Fig. 2). Along that sequence, we identify a dam portfolio that could have generated the same amount of energy as the current dam portfolio (Fig. 2A, “Optimal alternative to current portfolio”) while allowing 100 Mt/year instead of the current 52 Mt/year of sediment to reach the delta. That difference results from a different sequencing (Fig. 2C, blue markers) compared to the past and future BAU (Fig. 2B, blue markers). Most notably, dam sites further upstream on the Lancang, which have not yet been built and are not planned for several decades, would have been developed early on in the PSP sequence. Existing dams on the lower Lancang, such as Manwan Dam, would have been built later, allowing sediment continuity to persist for longer (compare the color code of dam sites between Fig. 2, B and C). In the PSP sequence, major dams that are now operational or nearing completion, such as Xayaburi and Lower Se San 2, would have been constructed only at the very end of the hydropower development process.

### Identifying limits for hydropower expansion through strategic planning

The optimal sequence for a pristine basin cannot be realized any longer because the already existing dams are incompatible with it. However, strategic planning in the basin from the current dam portfolio forward could still identify future dam development sequences with better trade-offs relative to continuing the BAU development. To explore remaining opportunities, we derived two alternative dam sequences, referred to as the “Whole Mekong strategic planning” (WMSP) and “Lower Mekong strategic planning” (LMSP) sequences (Table 1). The difference between the two sequences is that the WMSP sequence considers all potential remaining dam sites in the basin (both China and the lower Mekong), while the LMSP sequence considers only dam sites in the lower Mekong, i.e., where most of the hydropower expansion is planned in the near future.

**Table 1:** Overview over current and planned dam sequences and alternatives for strategic dam planning in the Mekong Basin.

**Dam** **sequences**	**Approach and decision variables** **(dams sites included in the optimization)**	**Comments**
BAU	Modeling the observed past and scheduled future dam sequence	Result of site-by-site development. No consideration of cumulative dam impacts and limited transboundary coordination.
PSP	All 124 dam sites in the basin considered as decision variables	Hypothetical sequence that could have been pursued if strategic planning had been adopted before the construction of any dams.
WMSP	68 undeveloped dam sites in the whole basin considered as decision variables	Strategic planning for the whole basin, i.e., including China and the lower Mekong. In reality, this would require mechanisms for coordination and benefit sharing between the lower basin countries and China.
LMSP	53 undeveloped dam sites in the lower basin considered as decision variables	Strategic planning focused member states of the MRC in the lower Mekong.

**Fig. 3:**
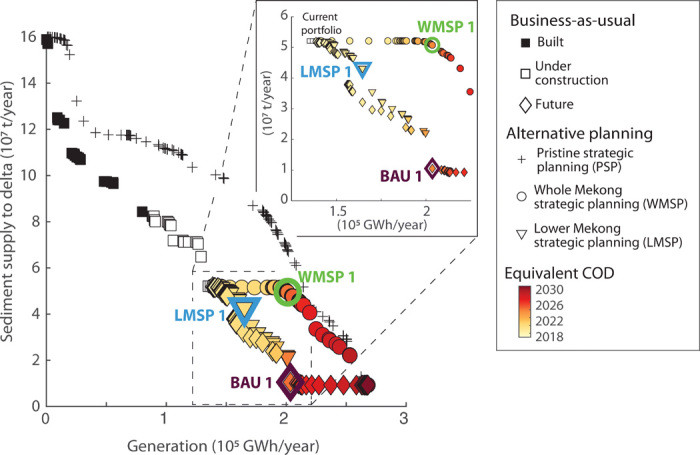
Remaining opportunities for strategic hydropower planning in the Mekong. Trade-offs between sediment supply to the delta and hydropower generation for the BAU sequence and two alternatives, WMSP (circles) and LMSP (triangles) (see Table 1 for details). The hypothetical dam trajectory for a pristine basin (PSP, compare Fig. 2) is shown for comparison (crosses). The inset emphasizes differences in planning alternatives starting from the current portfolio. Highlighted points along the three dam sequences (BAU 1, WMSP 1, and LMSP 1) are discussed in more detail in the text, and the resulting dam sequences and impact on network-scale sediment transport are shown in Figs. 4 and 5.

**Fig. 4:**
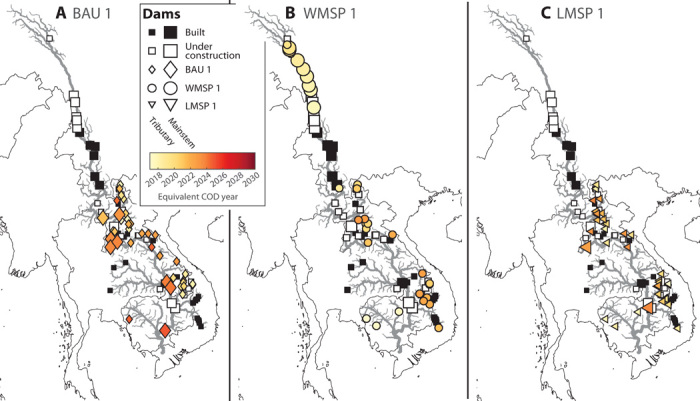
Spatiotemporal dam sequences for different planning strategies. Colored markers show the additional dams included in each of the three dam portfolios BAU 1 (**A**), WMSP 1 (**B**), and LMSP 1 (**C**) identified in Fig. 3. The color of each marker in (A) to (C) indicates the sequence with which dams in each portfolio would be constructed. White and black square markers indicate dams that are built or under construction included in all sequences.

The WMSP sequence (Fig. 3, round markers) shows a clear inflection point (Fig. 3, green circle marker). We designate the dam portfolio at this inflection point as WMSP 1. Adopting an optimal development sequence up to WMSP 1 would result in a marginal decrease in sediment supply to the delta of around 2 Mt/year down to 50 metric tons/year, a decrease of 4% compared to the present situation, and yield a 45% increase in generation. By contrast, achieving the same level of power generation with a portfolio along the BAU sequence would reduce sediment transport to the delta to 11 Mt/year, a decrease of 79% compared to the present situation (Fig. 3, violet diamond marker, hereafter referred to as BAU 1). In contrast to BAU 1 (Fig. 4A), WMSP 1 prioritizes dam construction in parts of the basin that are already disconnected from the delta, notable in the upper Lancang and in tributaries upstream of existing dams (Fig. 4B, light red markers). Dams on the lower mainstem of the Mekong, which is still largely connected to the delta, are not included in the WMSP 1 portfolio.

The LMSP sequence considers only dam sites in the lower Mekong and leads to trade-offs that are much closer to those under the BAU (Fig. 3, inset). There is no clear inflection point along the LSMP sequence, such as that observed for the WMSP sequence or as demonstrated in other studies on strategic hydropower planning (*18*, *20*). The absence of such an inflection point and the similarity of the BAU and LMSP sequences reflect the limited opportunities to expand hydropower in the lower basin with low impact. However, if the first major reduction in supply compared to the current portfolio was considered as the acceptable limit for expanding hydropower along the LMSP sequence, the resulting portfolio (LMSP 1; Fig. 3, blue triangle) would increase generation to 165,100 GWh/year (an increase of 19.3%) and reduce sediment supply to the delta to around 42 Mt/year, a 19% decrease compared to the present portfolio. A portfolio along the BAU sequence with the same generation would reduce sediment transport to the delta to around 30 Mt/year, a reduction of 42% from current levels. The LMSP 1 portfolio contains mainly dams on tributaries upstream and downstream of existing dams and two mainstem dams (those with the lowest marginal sediment trapping) (Fig. 4C).

Thus far, our analyses have focused on hydropower impacts on the sediment supply to the Mekong Delta. However, the effects of reduced sediment supply will affect river channels throughout the entire basin, with a range of impacts on geomorphic processes. For example, erosion and scouring of the riverbed are likely to occur in the alluvial reaches of the lower Mekong as a response to reduced sediment supply from upstream. The resulting channel incision and consequent lowered water levels, compounded by reduction in peak flows from flow regulation by upstream dams (*15*), will decrease lateral connectivity between river channels and floodplains, including flows into the Tonle Sap system. This loss in lateral connectivity may affect water and nutrient delivery to flood-recession agriculture and reduce access of fish to inundated floodplains that are essential spawning and rearing habitats for many species. Thus, these more distributed impacts create another important feedback between changing sediment budgets and livelihoods (*9*, *30*). Throughout the basin, bed and bank erosion in alluvial sections of the river might temporarily replace part of the sediment trapped behind dams. However, the availability of erodible bed material has already been reduced by widespread sand mining (*31*). The replacement would also be only temporary until in-channel sediment stores are depleted. Hence, in the CASCADE model, we do not account for this transient effect.

**Fig. 5:**
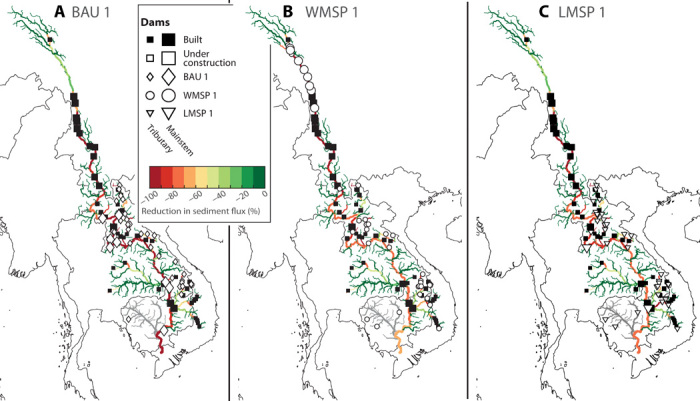
Impacts of different hydropower futures on network sediment flux. Results are shown for the BAU 1 (**A**), WMSP 1 (**B**), and LMSP 1 (**C**) portfolios (Fig. 3). Square markers in (A) to (C) mark dam sites that are built or under construction. Diamond (A), circle (B), or triangle (C) markers mark dam sites included in BAU 1, WMSP 1, and LMSP 1, respectively. The color of the river networks shows the relative reduction in sediment flux compared to pristine conditions without dams.

The altered sediment flux in the entire river network is shown in Fig. 5 for the different development sequences. Compared to a pristine basin, the BAU 1 portfolio reduces the sediment flux by more than 90% in most of the lower Mekong. Sediment flux in the upper Lancang would be reduced by around 30%. In contrast, the WMSP 1 portfolio would greatly fragment the upper Lancang and result in a reduction of sediment flux in most of the Lancang by 80% or more. However, the reduction in sediment flux in the lower Mekong and especially in Cambodia and Vietnam would be only 50 to 70%. This reduction is still large but much less compared to that under the BAU 1 portfolio. Focusing future dam construction principally on the lower basin, the LMSP 1 portfolio would maintain sediment flux in the upper Lancang at the current level and limit the reduction in the lower Mekong to no more than 70%. Some tributaries of the lower Mekong would experience a further reduction in sediment flux. However, that reduction would be mostly upstream of existing dams.

We introduce the sediment reduction index (SRI) to aggregate the impacts of dams on river sediment budgets in the different countries and compare hydropower impacts and generation on a national scale. The SRI (Materials and Methods) measures the average reduction of sediment transport over the river network in each country weighted by the area of affected riverbed. This weighting thus accounts for the extent of potentially affected aquatic and riparian habitat. Weighting by active channel area rather than simply length yields an index that is more comparable across the river network given the wide range of river channel sizes in different countries (*32*). For example, a certain percentage reduction in sediment flux in wide alluvial reaches in Cambodia and in the Vietnamese delta is likely to have a greater impact than the same percentage reduction in narrower headwater reaches (see the Supplementary Materials). The SRI ranges from 0 (no change) to 1 (all rivers in a country experience a 100% reduction of sediment flux).

**Fig. 6:**
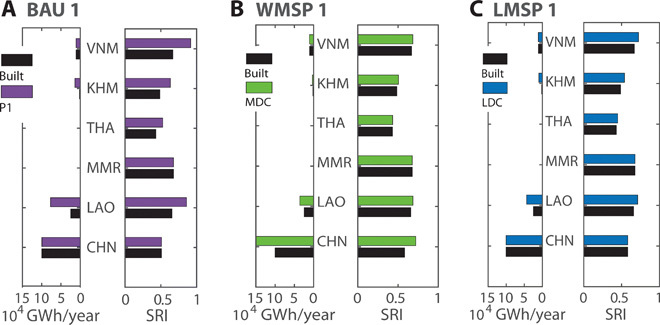
Share of hydropower impacts and benefits for different hydropower portfolios. Impacts on the river sediment budget of each country are measured as SRI for the BAU 1 (**A**), WMSP 1 (**B**), and LMSP 1 (**C**) portfolios and compared to changes in each country’s hydropower generation (see Fig. 5, A to C, for dams in each portfolio). Country labels are as follows: VNM, Vietnam; KHM, Cambodia; THA, Thailand; LAO, Laos; MMR, Myanmar; CHN, China. Note that Myanmar has no dam sites.

The BAU 1 portfolio results in a sixfold increase in Cambodia’s generation (1900 to 13,800 GWh/year) and an even larger absolute increase for Laos (24,600 to 77,800 GWh/year) in the next decade (Fig. 6A). The BAU 1 portfolio reduces sediment flux in the lower Mekong mainly because of the construction of major dams on the lower Mekong mainstem (Fig. 5A). BAU 1 changes the SRI from 0.58 to 0.7 as average over all countries, with a large change in Laos and Cambodia (Laos, 0.66 to 0.86; Cambodia, 0.49 to 0.63), hence where most of the additional hydropower would be developed (Fig. 6A). Vietnam would experience an even larger increase in SRI (0.67 to 0.92) without any additional direct hydropower generation, although Vietnam might import power from planned dams.

The WMSP 1 portfolio would shift generation and dam impacts from the Mekong Delta to the largely undammed upper Lancang. Under WMSP 1, hydropower would be expanded to 35,800 GWh/year in Laos (+46%) and 3300 GWh/year (+70%) in Cambodia. Generation in China would expand from 100,200 to 150,300 GWh/year (+50%, Fig. 6B). The increase in SRI for WMSP is only 5% as average over all countries, but nearly all the impact is in the Chinese Lancang (from 0.58 to 0.72; Figs. 5B and 6B).

Compared to WMSP 1, for which the increase of hydropower generation and impacts is concentrated in China, LMSP 1 distributes additional impacts and hydropower generation more evenly among countries. The average SRI in the whole network is nearly identical for WMSP 1 and LMSP 1 (0.61 and 0.62, respectively). However, in contrast to WMSP 1, no country experiences an increase of SRI of more than 11% for the LMSP 1 portfolio (Laos, +8%; Vietnam, +8%; Thailand, +4%; Cambodia, +10%; China, ±0%). This LMSP 1 increase is less than the >30% increase in SRI for most of the lower basin countries under the BAU 1 portfolio. For the LMSP 1 portfolio, most of the total added generation 26,700 GWh/year is in Laos (+18,700 GWh/year) and Cambodia (+7800 GWh/year) (Fig. 6C).

To conclude, the WMSP 1 leads to an extreme concentration of hydropower impacts and generation in China and shifts the future expansion of hydropower from the lower to the upper basin. This is challenging in the current political setting of the basin given the power demand of LMB countries and their interest in revenues from power exports. In contrast, the LMSP 1 portfolio more evenly distributes hydropower impacts and hydropower generation over the basin and does still result in a major increase in hydropower generation in Laos and Cambodia.

## DISCUSSION

Our findings provide guidance for future hydropower development in the Mekong and showcase methodological advances for planning more sustainable hydropower in other basins with ongoing hydropower development. The approach used here applies a simplified representation of natural processes on a basin scale. The resulting screening-level analysis of different development policies provides insights that can be integrated into multiscale, multiobjective, and multidomain analyses in the future.

Numerically effective tools like CASCADE are crucial for basin-scale studies of river processes, screening and optimizing different development alternatives, and analyzing robustness and sensitivity to poorly monitored river processes such as sediment transport. This is relevant for large rivers such as the Mekong, where data on sediment transport are scarce and fragmented between different countries. Our results show that trade-offs improve at larger planning scales, from individual dams, to the lower basin, and to the whole basin, a finding which is in agreement with spatial design principles for dam portfolios more generally (*12*). Hence, taking a basin-scale perspective even in the face of data scarcity is crucial.

Screening models operating at large scales and transboundary settings will typically be limited in their capacity to represent processes because of lack of data and/or because of trade-offs between model complexity and the computational efficiency required for optimization-based approaches. For this study, these limitations include the inability to account for morphologic processes, such as riverbed erosion, which might temporarily buffer dam impacts on the basin’s sediment budget (Supplementary Method 2). The model also includes neither impacts of flow regulation on sediment transport nor possible management strategies to improve sediment passage through dams (Supplementary Method 3) (*25*, *33*).

An actual application of basin-scale strategic planning for the Mekong should be expanded to include additional objectives, such as hydrologic alterations (*34*), impacts on terrestrial ecosystems, and impacts on fish migration and aquatic biodiversity (*20*). For example, by focusing on the single environmental objective of sediment, the LMSP 1 portfolio contains two dams on the Mekong (Stung Treng and Sanakham) that have relatively low impacts on sediment but would have major impacts on fish migration (*20*), protected wetlands, and some of the last refuges for critically endangered dolphins (*10*). Hence, real-world application of strategic planning will need to identify other critical trade-offs among environmental objectives and include them into the planning process. Ensuring that the derived dam sequences are economically feasible will require considering additional economic and operational objectives, e.g., generation costs, storage capacity of resulting dam portfolios, and its peaking capacity (*18*, *19*, *21*).

Coupling planning approaches across scales, from dam portfolios on basin scales to single dams, is another key research priority. At the scale of individual dams, optimizing operation rules and designs for multiple objectives can reduce impacts and could thus improve trade-offs across dam portfolios (*25*, *33*). Studying designs of mainstem dams still included in the LMSP 1 portfolio could identify if there are options for lower impact redesigns of these dams. In turn, studies on the scale of single dams (*25*) would benefit from an integration in a basin-scale strategic vision, e.g., to identify alternatives to dams whose sites are inherently problematic or for which a redesign is economically or technically not feasible.

Despite uncertainties, our results indicate that opportunities remain to improve trade-offs between future hydropower development and environment in the Mekong Basin. Planning on the basin scale could minimize impacts in the lower basin countries by concentrating future hydropower in the Lancang above large existing dams. However, such a strategy is in conflict with the ambition of the countries in the lower basin to expand hydropower generation as an export commodity (*11*) and would concentrate all environmental impacts in China. A dam portfolio that considers options in the lower basin only, such as LMSP 1, results in much less generation and has higher impacts but is potentially more realistic. However, strategic dam planning would require increased coordination between lower basin countries. This could be achieved if existing organizations such as the MRC were strengthened and if emerging whole-basin organizations such as the Lancang-Mekong Cooperation embraced strategic hydropower planning. Transparent sharing of discharge and sediment data as basis for decision-making would be an important first step in that direction.

Beyond strategic hydropower planning, integrated energy planning that considers a full range of renewable technologies (photovoltaic, wind, hydropower, and biomass) has a major potential to provide practical renewable energy solutions for emerging economies (*26*, *27*). A recent study suggested that the lower Mekong region could meet future electricity demands with considerably less hydropower than anticipated under a BAU trajectory, mostly because of dropping prices of other renewables (*35*). This study anticipated some additional hydropower generation in the basin (around 25,000 GWh/year), roughly comparable to that provided by the LMSP 1 portfolio. A slight decrease in that level of hydropower expansion (e.g., to 22,000 GWh/year) through improved efficiency or additional investment in solar photovoltaic would allow the exclusion of the high-impact Stung Treng dam from the LMSP 1 portfolio.

Rethinking the planning of hydropower within renewable energy systems is a global challenge for river basin management. Globally, there are around 3700 potential future dam sites, many in vulnerable basins of the last free-flowing rivers, such as the Amazon, Salween, and Irrawaddy (*1*, *36*). Governance is weak or absent in most of these transboundary basins, with a notable absence of mechanisms to strategically plan infrastructure investments, and dam development mostly proceeds project-by-project. Our results from the Mekong provide clear evidence that project-by-project development will generally produce suboptimal trade-offs and an unequitable sharing of impacts and benefits among riparian countries. In contrast, strategic hydropower planning can identify development pathways that minimize impacts while delivering energy benefits within broader renewable energy strategies.

## MATERIALS AND METHODS

### Collecting dam data

We collected information on 124 dams in the basin from published dam databases (*4*, *5*, *37*). The model required some key parameters for each dam site: (i) location; (ii) mean annual inflow (m^3^/year); (iii) total storage, i.e., sum of dead and live storage (m^3^); (iv) annual generation (GWh/year); and (v) status and commercial operation data (COD).

For sites with any of these parameters missing, we supplemented the tabulated data via interpolation from other dam sites or from global geospatial data, as described in Supplementary Method 1 and figs. S1 and S2. The database notably omits smaller hydropower dams and many irrigation dams, especially in Thailand, and their potential impacts (*38*). However, the database, available as a supplementary Excel file (data S1) presents the largest available set of dams with consistent availability of relevant data.

### Finding PO dam portfolios

To derive PO dam portfolios for the whole Mekong Basin, we used the Borg multiobjective evolutionary algorithm (*24*) to solve a decision problem of the form

$$\underset{u}{\text{min}}(-J_{1}(u),-J_{2}(u))$$

In which *J*_1_ and *J*_2_ are the indicators for (i) hydropower generation (GWh/year) and (ii) sediment supply (tons of sediment supply to the delta per year), respectively. *u* is a binary decision vector, consisting of 1…*N*_sites_ binary decision variables, that indicates whether a dam site is selected as part of a portfolio. The optimization identified PO portfolios *P*_PO_, i.e., portfolios with an optimal trade-off between the two considered objectives.

### Indicator for hydropower generation

We calculated the annual generation of portfolio *P*, *J*_1_(*P*) (GWh/year) as sum of tabulated mean annual values, *W*, of all dams in portfolio *P*

$$J_{1}(P)=\sum_{d\in P}W(d)$$

*W* is available from tabulated data for dams in the Lower Mekong River Basin and the Lancang (*4*, *5*, *37*). If data on generation were missing, we interpolated generation from the tabulated installed capacity (see Supplementary Method 1 and fig. S2).

### Indicator for sediment supply to the Mekong Delta

Calculating the sediment supply to the Mekong Delta for a dam portfolio *P* required information on natural sediment transport in the river network and on sediment trapping in each dam. On the basis of this information, we calculated the cumulative trapping in all dams belonging to a portfolio and its reciprocal, the residual sediment supply to the delta. We represented the natural sediment transport to the Mekong Basin using a combination of the CASCADE framework for sediment supply (*23*) combined with distributed geomorphic estimates for sediment yield in the Mekong Basin (*8*). More details on model formulation and a model definition sketch are available from Supplementary Method 2 and figs. S3 and S4.

The procedure is as follows: Kondolf *et al.* (*8*) divided the Mekong Basin into nine geomorphic provinces based on topography, climate, tectonic history, and lithological parameters. They assigned a specific sediment yield (t/km^2^/year) to each geomorphic province such that the total load at the basin outlet matched an estimated 160 Mt/year (fig. S3). However, Kondolf *et al.* did not include an explicit network-scale routing scheme. We hence combined the sediment yield estimate from Kondolf *et al.* with the CASCADE framework for sediment routing.

We first derived the drainage area and the river network from a 250-m resolution digital elevation model (DEM) (*39*) using standard procedures (*40*). Because the drainage area derived from the DEM is ca. 10% larger than the value that was used by Kondolf *et al*. (*8*), we scaled the values from Kondolf *et al.* such that the sediment supply to the Mekong Delta was 160 Mt/year (fig. S3). We assigned a sediment source to each reach in the network and calculated the sediment supply rate of each source from the direct drainage area and the sediment yield of the geomorphic province in which a reach was located.

The pristine sediment supply to the delta is represented by the sediment flux in the most downstream reach, denoted as Ω, and is calculated as

$$\Theta_{S,\Omega }=\sum_{Ϛ\in \Gamma \Omega }\;\Theta_{S,\Omega }^{Ϛ}$$

where Γ_Ω_ is the set of all sediment cascades, i.e., of all processes delivering the sediment from all upstream sources to the basin outlet. $\Theta_{S,\Omega }^{Ϛ}$ is the sediment supply (t/year) from a specific sediment source, ς, to the delta. One or multiple dams between a sediment source ς and Ω reduce the sediment supply according to the trap efficiency, *TE*, of the dams. We defined *D*(ς, Ω, P) as the set of dams that is added between source ς and the basin outlet Ω as part of dam portfolio *P*.${\Theta_{S,\Omega }^{Ϛ}}^{*}(P)$ is the resulting modified sediment supply from ς to the delta.${\Theta_{S,\Omega }^{Ϛ}}^{*}(P)$ is defined as

$${\Theta_{S,\Omega }^{Ϛ}}^{*}(P)=[\prod_{k\in D(Ϛ,\Omega ,P)}(1-{\mathrm{TE}}_{k})]*\Theta_{S,\Omega }^{Ϛ}$$

We then calculated the total sediment supply to the delta for portfolio *P* as

$$J_{2}(P)=\sum_{Ϛ\in \Gamma \Omega }{\Theta_{S,\Omega }^{Ϛ}}^{*}(P)$$

i.e., the modified sediment supply from all sources to the delta. *J*_2_(*P*) was used as second objective in the Borg algorithm. The same approach was used to simulate the sediment trapping for the past and BAU development of dams (fig. S5).

We calculated the sediment trap efficiency in each dam using the Brune model (*41*), an empirical model based on the hydraulic residence time of each dam’s reservoir. We used the Brune model because of its limited data demand, which makes it suitable for data-poor settings, such as the Mekong, and for consistency with previous regional studies (*8*, *14*, *42*). The Brune model was conceived for suspended sediment only, while the Mekong transports a relevant fraction of (sandy) bedload (*31*). We corrected the Brune trap efficiency assuming that each reach transports 10% of the total load as sand (*43*), which is completely trapped behind dams (Supplementary Method 3). The resulting model evaluated the impact of a given dam portfolio in less than a second on a personal computer, which resulted in a runtime of few hours for a basin-scale optimization.

### Deriving optimal dam sequences

The objective of optimal dam sequencing was not only to derive PO dam portfolios but also to determine the sequence with which dams should be built to match a certain future hydropower demand. Optimal dam sequences are adaptive in the sense that development can be stopped, e.g., if there is less demand than projected, and the resulting dam portfolio will still create good trade-offs. Some conceptual examples for the benefits of dam sequencing are shown in figs. S6 and S7. To derive dam sequences, we calculated with which probability ${𝒫}_{d}$ a dam site d occurs in any PO portfolio, i.e., ${𝒫}_{d}(d\in P_{\mathrm{PO}})$. Second, we sorted all dam sites according to ${𝒫}_{d}$. This resulted in a rank $R_{d}$ of each site d in the sequence so that $R_{d}=1$ is, e.g., the first ranking site. We then arranged the dams such that

$$R_{d}=1,R_{d}=2,\ldots ,R_{d}=N_{\text{Sites}}-1,R_{d}=N_{\text{Sites}}$$

so that the order of subscripts *d* would identify the optimal dam sequence. We assigned an equivalent COD year to each dam in that sequence based on the COD of a point along the BAU sequence with a comparable generation. For example, in Fig. 3, the BAU 1 portfolio presents the status of the basin by around 2025 according to tabulated information and will generate 200,000 GWh/year. The alternative portfolio WMSP 1 provides the same amount of hydropower, and we hence assume that its equivalent COD would be 2025. We hence implicitly assumed that the planned expansion of hydropower in the basin is representative for the actual growth in demand. However, it should be noted that the actual future demand might change according to the economic growth and availability and costs of alternative renewables.

Last, we compared the trade-offs along an optimal sequence with the trade-offs created by individual portfolios along a Pareto frontier. We evaluated how sequences derived from an alternative greedy algorithm performed compared to the proposed sequencing algorithm (Supplementary Method 5 and Supplementary Result 1). We found that the Pareto sequencing creates trade-offs that are closer to the Pareto frontier, especially for higher generation. We hence used Pareto sequencing throughout this paper (Supplementary Result 1 and figs. S9 and S10). Supplementary Method 5 and figs. S5 to S7 give a detailed description, rationale, and additional visualizations of the sequencing approach. Supplementary Result 1 and figs. S9 and S10 give the results of the greedy algorithm.

### Sediment reduction index

The CASCADE model represents river reaches as edges in a directed graph. Let *e* be any edge in the river network and ς a sediment source. Then, the model calculates the incoming sediment flux in the natural state as

$$\Theta_{S,e}=\sum_{Ϛ\in \Gamma e}\Theta_{S,e}^{Ϛ}$$

where Γ*_e_* is the set of all sediment cascades connecting edge *e* to upstream sediment sources. The sediment flux to *e* for a specific dam portfolio is then

$${\Theta_{S,e}}^{*}(P)=\sum_{Ϛ\in \Gamma e}{\Theta_{S,e}^{Ϛ}}^{*}(P)$$

The relative change in flux for portfolio *P* in edge *e* is defined as

$$\Delta \Theta_{S,e}(P)=\frac{\Theta_{S,e}*(P)-\Theta_{S,e}}{\Theta_{S,e}}$$

We aimed to calculate an SRI, SRI*_c_*(*P*), for a riparian country *c* for portfolio *P*. For that, we first determined the set of all edges in country *c*, *E_c_*. Then, we calculated the SRI*_c_*(*P*) as the sum of change in sediment flux for portfolio *P* in *E_c_*, weighted by the area of riverbed, *A*_AC_, affected

$$\text{SR}I_{c}(P)=\frac{\sum_{k\in E_{c}}\Delta \Theta_{S,k}(P)*A_{\text{AC},k}}{\sum_{k\in E_{c}}A_{\text{AC},k}}$$

This indicator considers for how much of the active channel area of a nation is affected by a reduction in sediment flux for a dam portfolio.

We calculated the bed area of each edge from

$$A_{\text{AC},e}=W_{\text{AC},e}*L_{e}$$

where *W*_AC,*e*_ is the active channel width and *L_e_* is the length of edge *e*. *L_e_* was a direct result when extracting the river network from the DEM (*40*), and we derived *W*_AC,*e*_ using a regression based on geomorphic attributes (*23*) (Supplementary Method 6).

## Supplementary Material

http://advances.sciencemag.org/cgi/content/full/5/10/eaaw2175/DC1

Download PDF

data S1

Planning dam portfolios for low sediment trapping shows limits for sustainable hydropower in the Mekong

## SUPPLEMENTARY MATERIALS

Supplementary material for this article is available at http://advances.sciencemag.org/cgi/content/full/5/10/eaaw2175/DC1 Fig. S1. Deriving missing inflow data for dams in the database. Fig. S2. Deriving missing generation data for dams in the database. Fig. S3. Delineating geomorphic provinces and sediment flux in the Mekong Basin. Fig. S4. Definition sketch of the CASCADE model. Fig. S5. Modeling trade-offs along a past and planned dam development sequence. Fig. S6. Conceptualizing challenges for deriving an optimal dam sequence from optimal dam portfolios. Fig. S7. Deriving optimal development sequences from PO dam portfolios. Fig. S8. Greedy algorithm for dam sequencing. Fig. S9. Comparing BAU and different algorithms for dam sequencing to optimal dam portfolios. Fig. S10. Spatiotemporal dam sequences from different algorithms. Supplementary Method 1. Building the dam database Supplementary Method 2. Detailed description of the CASCADE model Supplementary Method 3. Calculating trap efficiencies Supplementary Method 4. Quantifying performance of past and future dam sequences Supplementary Method 5. Transferring optimal dam portfolios into optimal dam sequences Supplementary Method 6. Deriving active channel width Supplementary Result 1. Performance of different sequencing algorithms Coordinates and characteristics of all dam sites included in this analysis are available in data S1 provided with this paper. Additional data related to this paper may be requested from the authors. References (*44*–*52*)
