# Supplementary material for: Planning dam portfolios for low sediment trapping shows limits for sustainable hydropower in the Mekong
Source: Sci Adv. 2019 Oct 23;5(10):eaaw2175. doi: 10.1126/sciadv.aaw2175 (PMC6984971; doi:10.1126/sciadv.aaw2175)
Supplement: http://advances.sciencemag.org/cgi/content/full/5/10/eaaw2175/DC1 [file supp_5_10_eaaw2175__index.html]

Science Advances | Science AdvancesAAASSearchScience AdvancesMenu

## Supplementary Materials

**The PDF file includes:**

- Fig. S1. Deriving missing inflow data for dams in the database.
- Fig. S2. Deriving missing generation data for dams in the database.
- Fig. S3. Delineating geomorphic provinces and sediment flux in the Mekong Basin.
- Fig. S4. Definition sketch of the CASCADE model.
- Fig. S5. Modeling trade-offs along a past and planned dam development sequence.
- Fig. S6. Conceptualizing challenges for deriving an optimal dam sequence from optimal dam portfolios.
- Fig. S7. Deriving optimal development sequences from PO dam portfolios.
- Fig. S8. Greedy algorithm for dam sequencing.
- Fig. S9. Comparing BAU and different algorithms for dam sequencing to optimal dam portfolios.
- Fig. S10. Spatiotemporal dam sequences from different algorithms.
- Supplementary Method 1. Building the dam database
- Supplementary Method 2. Detailed description of the CASCADE model
- Supplementary Method 3. Calculating trap efficiencies
- Supplementary Method 4. Quantifying performance of past and future dam sequences
- Supplementary Method 5. Transferring optimal dam portfolios into optimal dam sequences
- Supplementary Method 6. Deriving active channel width
- Supplementary Result 1. Performance of different sequencing algorithms
- References (*44*–*52*)

Download PDF

**Other Supplementary Material for this manuscript includes the following:**

- Coordinates and characteristics of all dam sites included in this analysis are available in data S1 provided with this paper. Additional data related to this paper may be requested from the authors.

**Files in this Data Supplement:**

- Adobe PDF - aaw2175\_SM.pdf
