# Supplementary material for: Planning dam portfolios for low sediment trapping shows limits for sustainable hydropower in the Mekong
Source: Sci Adv. 2019 Oct 23;5(10):eaaw2175. doi: 10.1126/sciadv.aaw2175 (PMC6984971; doi:10.1126/sciadv.aaw2175)
Supplement: Download PDF [file aaw2175_SM.pdf]

Science Advances
AAAS

advances.sciencemag.org/cgi/content/full/5/10/eaaw2175/DC1

Supplementary Materials for

Planning dam portfolios for low sediment trapping shows limits for sustainable hydropower in the Mekong

R. J. P. Schmitt*, S. Bizzi, A. Castelletti, J. J. Opperman, G. M. Kondolf

*Corresponding author. Email: rschmitt@stanford.edu

Published 23 October 2019, *Sci. Adv.* **5**, eaaw2175 (2019)
DOI: 10.1126/sciadv.aaw2175

The PDF file includes:

Fig. S1. Deriving missing inflow data for dams in the database.
Fig. S2. Deriving missing generation data for dams in the database.
Fig. S3. Delineating geomorphic provinces and sediment flux in the Mekong Basin.
Fig. S4. Definition sketch of the CASCADE model.
Fig. S5. Modeling trade-offs along a past and planned dam development sequence.
Fig. S6. Conceptualizing challenges for deriving an optimal dam sequence from optimal dam portfolios.
Fig. S7. Deriving optimal development sequences from PO dam portfolios.
Fig. S8. Greedy algorithm for dam sequencing.
Fig. S9. Comparing BAU and different algorithms for dam sequencing to optimal dam portfolios.
Fig. S10. Spatiotemporal dam sequences from different algorithms.
Supplementary Method 1. Building the dam database
Supplementary Method 2. Detailed description of the CASCADE model
Supplementary Method 3. Calculating trap efficiencies
Supplementary Method 4. Quantifying performance of past and future dam sequences
Supplementary Method 5. Transferring optimal dam portfolios into optimal dam sequences
Supplementary Method 6. Deriving active channel width
Supplementary Result 1. Performance of different sequencing algorithms
References (*44–52*)

Other Supplementary Material for this manuscript includes the following:

(available at advances.sciencemag.org/cgi/content/full/5/10/eaaw2175/DC1)

Coordinates and characteristics of all dam sites included in this analysis are available in the Excel file data S1 provided with this paper. Additional data related to this paper may be requested from the authors.

Supplemental Figures

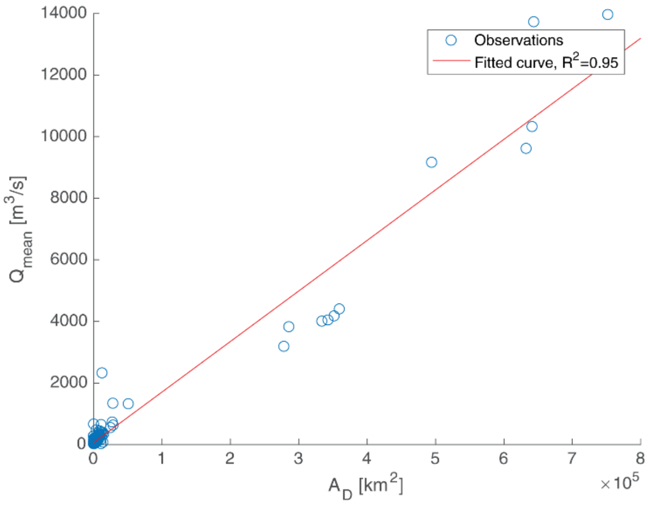

Fig. S1. Deriving missing inflow data for dams in the database. Inflow data are required for all dams to calculate sediment trap efficiencies. We derive a linear regression between the DEM-derived drainage area and mean inflow for all dams with tabulated inflow data. The regression resulted in a very good fit between drainage area and mean inflow (R2 = 0.95). We use the derived regression to calculate mean inflows for dams without these data.

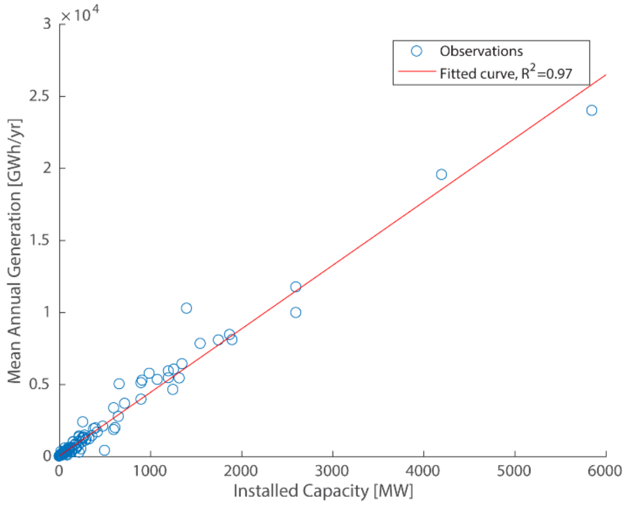

Fig. S2. Deriving missing generation data for dams in the database. Generation at a dam site can be estimated by a linear regression between installed capacity and tabulated annual hydropower generation. The regression resulted in a very good fit between installed capacity and generation (R^2 = 0.97). This regression is used to estimate generation for dam sites without tabulated information on generation.

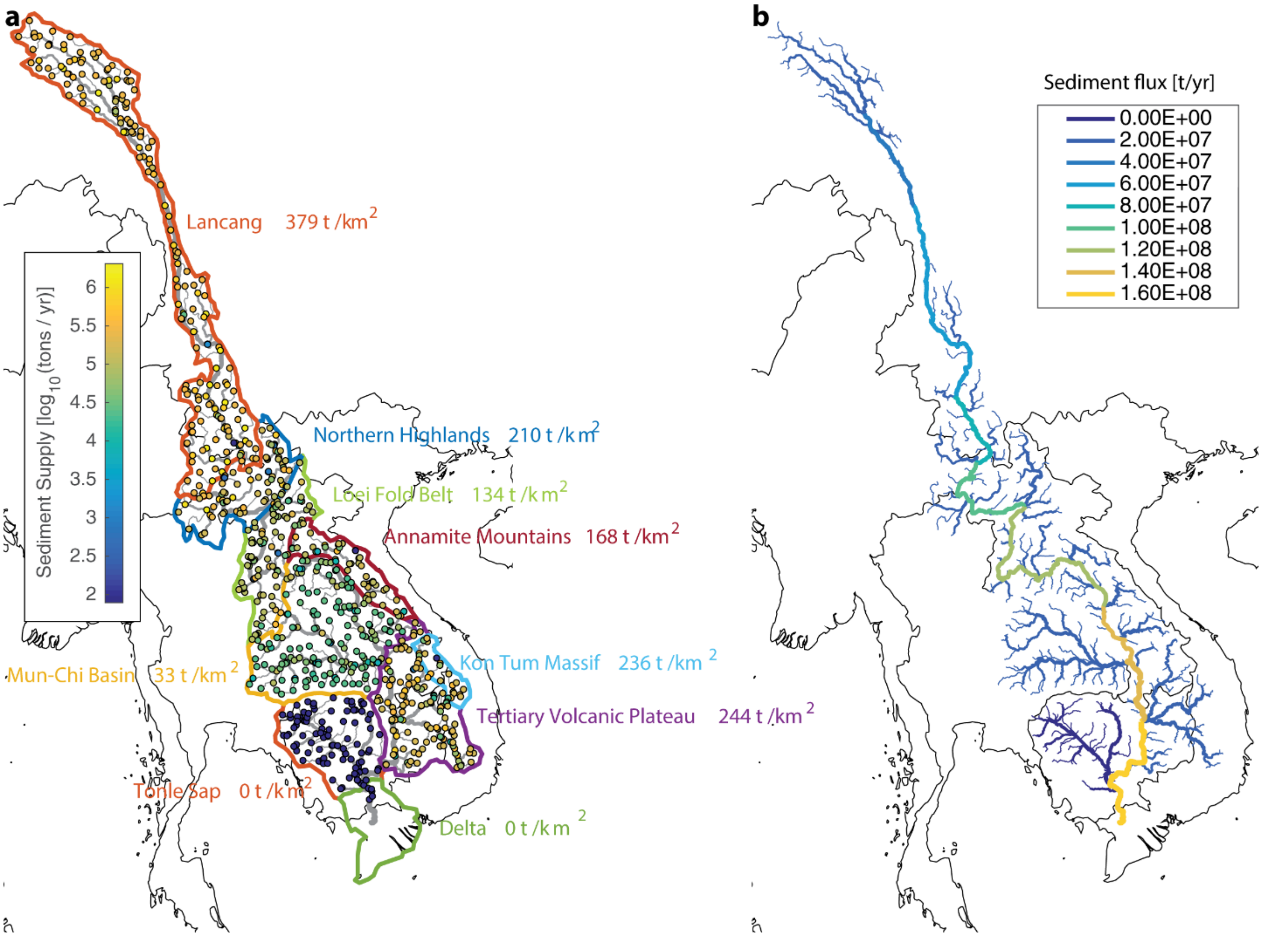

Fig. S3. Delineating geomorphic provinces and sediment flux in the Mekong Basin. Location of geomorphic provinces used as input to build the CASCADE model (see Methods and Supplemental Methods 2). Each province is assigned a specific sediment yield, y [t/km^2/yr] based on Kondolf et al. (2014) (8). Sediment sources (dots in panel a) are assigned a specific supply $\Theta_{S,in}^{\varsigma}$ [t/yr] (see colors) according to their location and their direct drainage area. The graph-based CASCADE routing scheme for sediment (23) is applied to route the sediment from each source to outlet of the network, resulting in information on sediment flux in the network (b shows pristine sediment flux without dams).

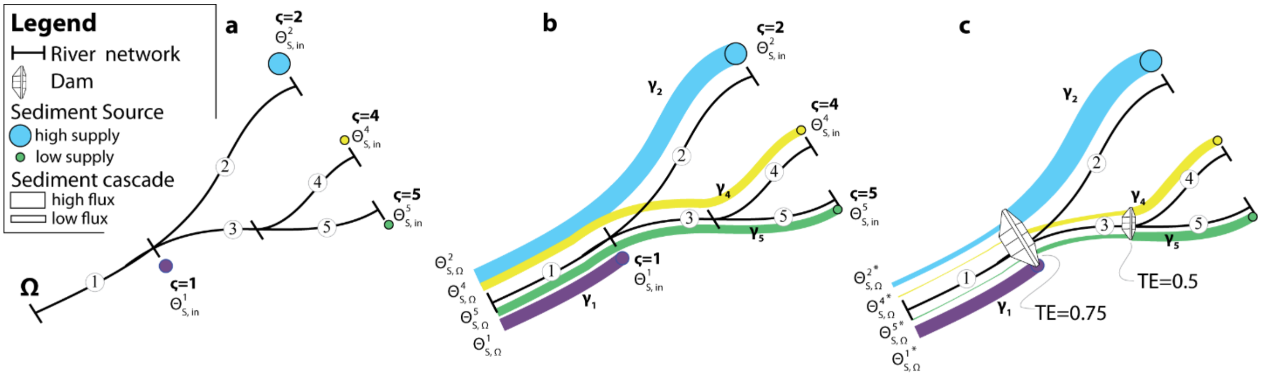

Fig. S4. Definition sketch of the CASCADE model. The model set-up is shown for a river network with $e = 1 \dots 5$ $e$dges and $\varsigma = 1 \dots 5$ sediment sources. a: defining the river graph and sediment source properties. b: deriving sediment flux and connectivity along multiple sediment cascades. c: changed magnitude and pattern of sediment flux after the addition of multiple dams.

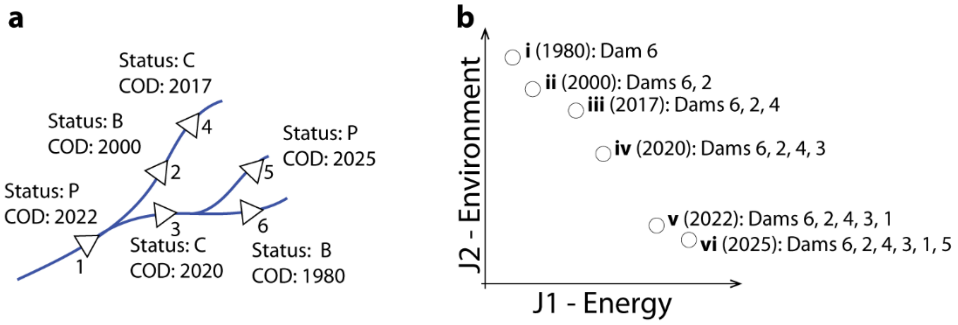

Fig. S5. Modeling trade-offs along a past and planned dam development sequence. Triangles with numbers identify dam sites. The sequence is based on the tabulated status (B: built, C: under construction, P: planned) and the commercial operation date (COD) of dams (a). The derived sequence allows simulating the development of trade-offs resulting from that sequence over time. Each portfolio in the sequence (roman numbers i-vi) can be assigned a time based on the COD of the last added dam.

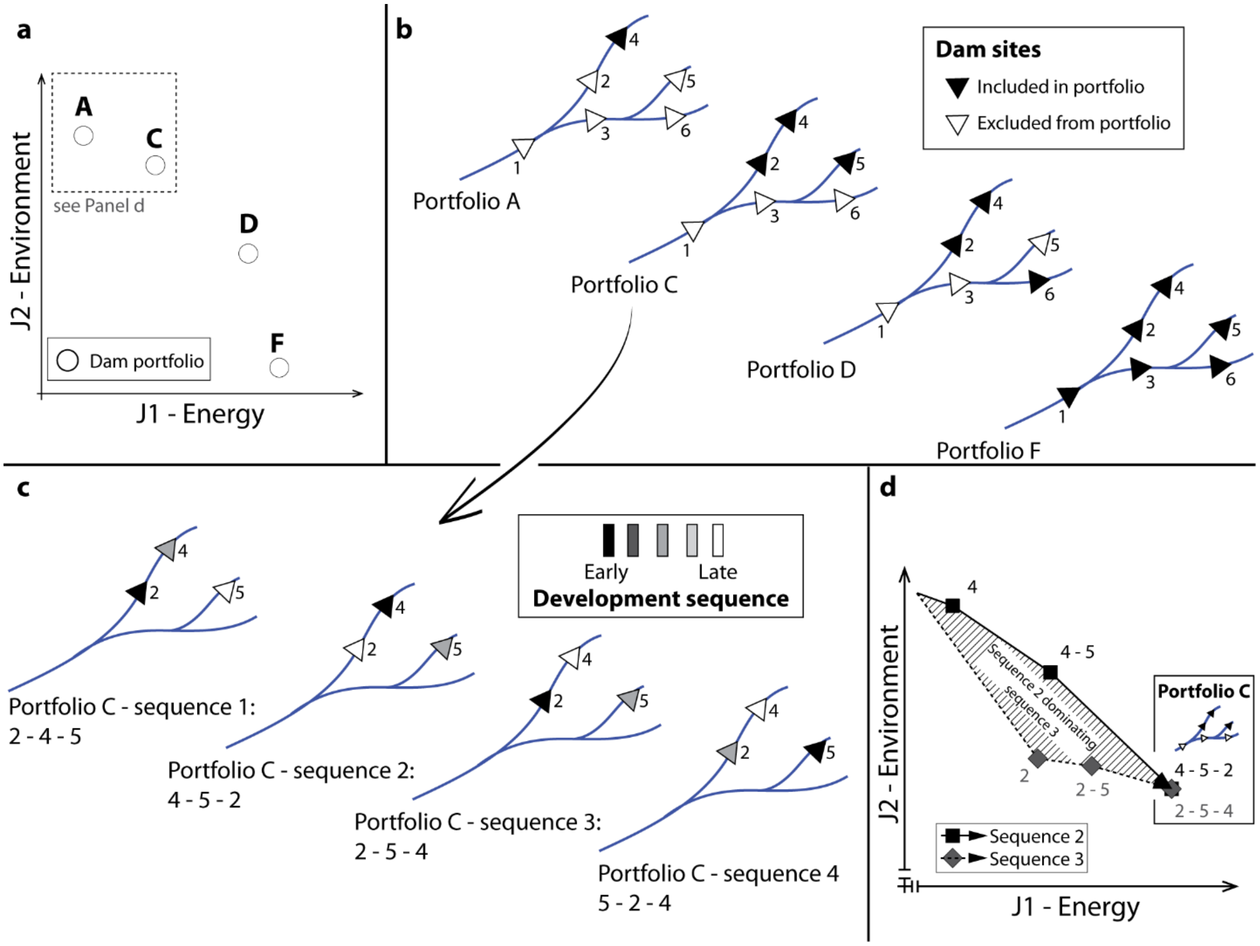

Fig. S6. Conceptualizing challenges for deriving an optimal dam sequence from optimal dam portfolios. A set of Pareto-optimal dam portfolios with increasing energy generation or installed capacity (a) does not necessary form a practical dam sequence (b). For example, Portfolio C and D do not form a sequence, as they contain different dam sites (black triangles in b). Even for a single portfolio, e.g., portfolio C, sites within that portfolio can be developed in different sequences (c). The colors of triangles from black to white indicate if a dam is developed early or late. Over time, each of the sequences shown in c would result in a different temporal development of trade-offs (d). Notably, sequence 2 (build small dams first) results in lower impacts for intermediate levels of development compared sequence 3 (build large dams first). While both sequences (2-5-4 vs. 4-5-2) result in the same final portfolio (i.e., portfolio C containing sites 2, 4, and 5) with the same trade-offs, sequence 2 creates a “no regret” strategy if dam development is terminated before all dams are build, as sequence 3 is dominated by sequence 2 (panel d).

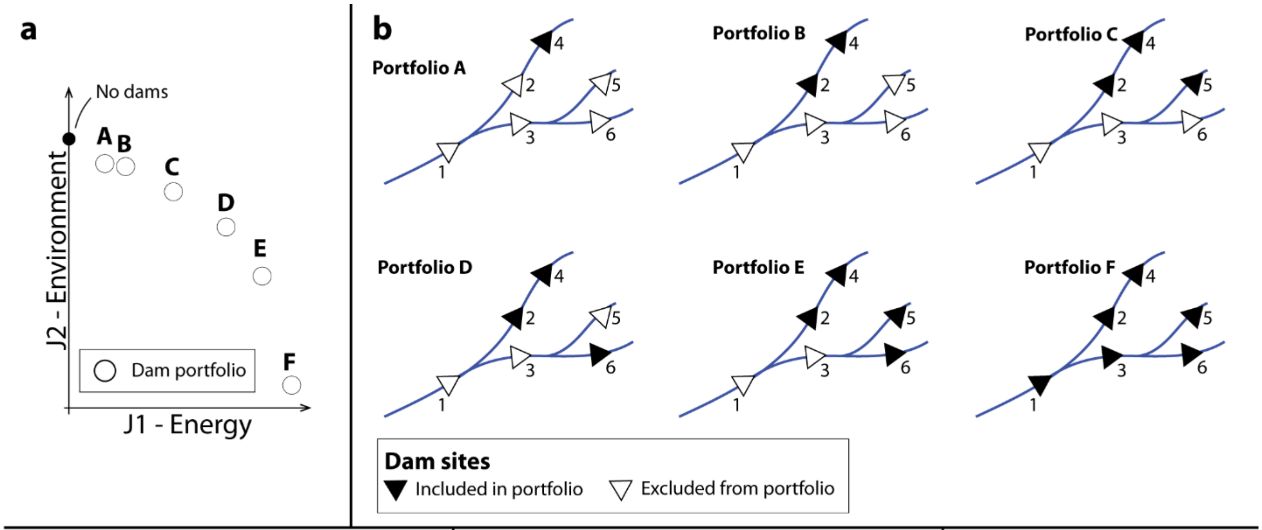

c

	Site 1	Site 2	Site 3	Site 4	Site 5	Site 6
Portfolio A	0	0	0	1	0	0
Portfolio B	0	1	0	1	0	0
Portfolio C	0	1	0	1	1	0
Portfolio D	0	1	0	1	0	1
Portfolio E	0	1	0	1	1	1
Portfolio F	1	1	1	1	1	1
**Probability of inclusion**	**17 %**	**83 %**	**17 %**	**100 %**	**50 %**	**50 %**

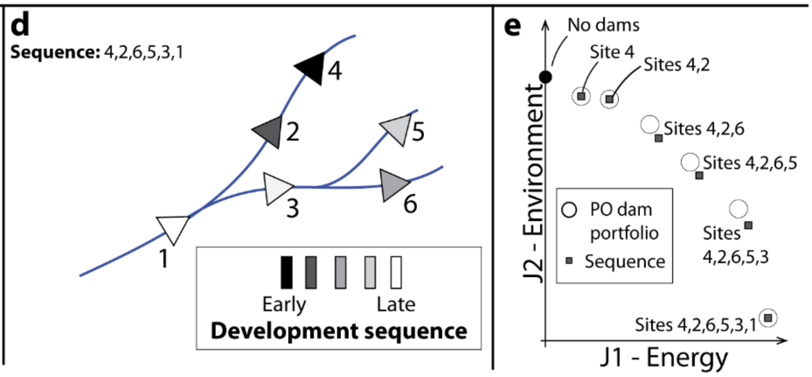

Fig. S7. Deriving optimal development sequences from PO dam portfolios. First, optimal dam portfolios are sorted according to their generation (a, capital letters). Each portfolio contains a specific set of dam sites (b). From the probability that a dam is included in any portfolio (c) we determine the optimal development sequence (d). e shows the simulation of the optimal development sequence (squares) compared to the PO Dam portfolio (circle).

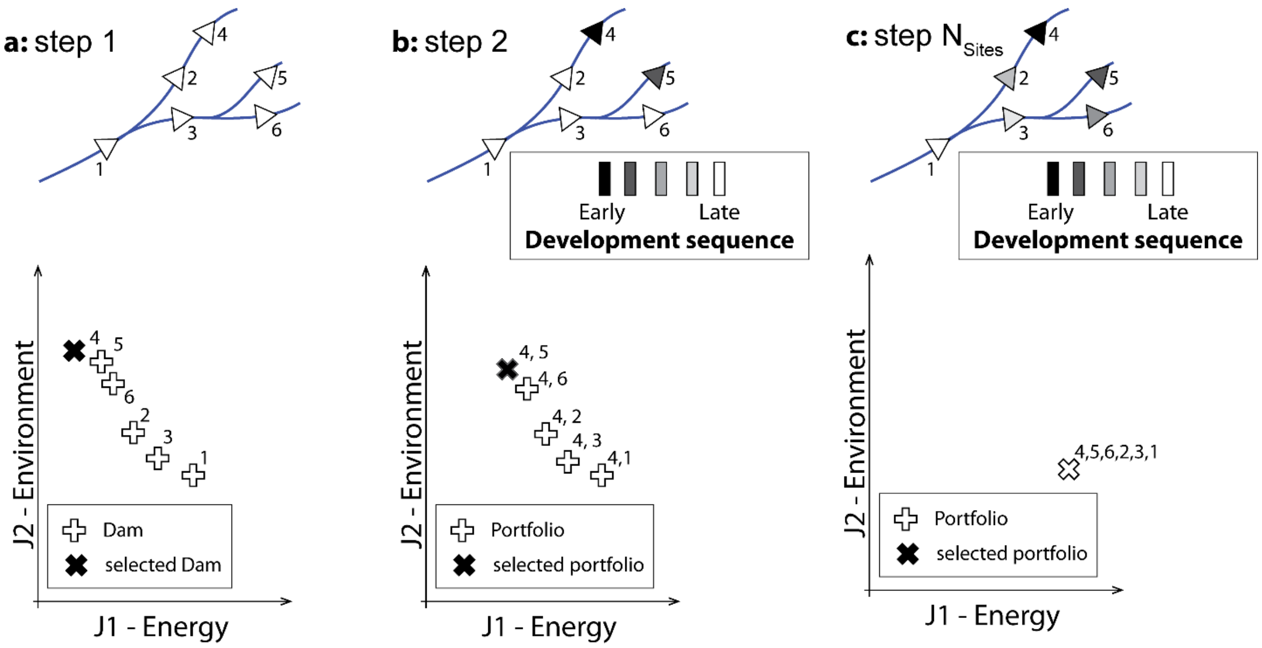

Fig. S8. Greedy algorithm for dam sequencing. In a first step, the performance of all dam sites is evaluated and the site with the lowest impact is selected (a). In the next step, all portfolios resulting from combining the dam selected in step 1 with each remaining dam site are evaluated and the portfolio with the next lowest impact is selected. This process is repeated until a final portfolio consisting of all dam sites is reached. The sequence (see color of triangles in c) is determined according to the step in which a specific site has been selected.

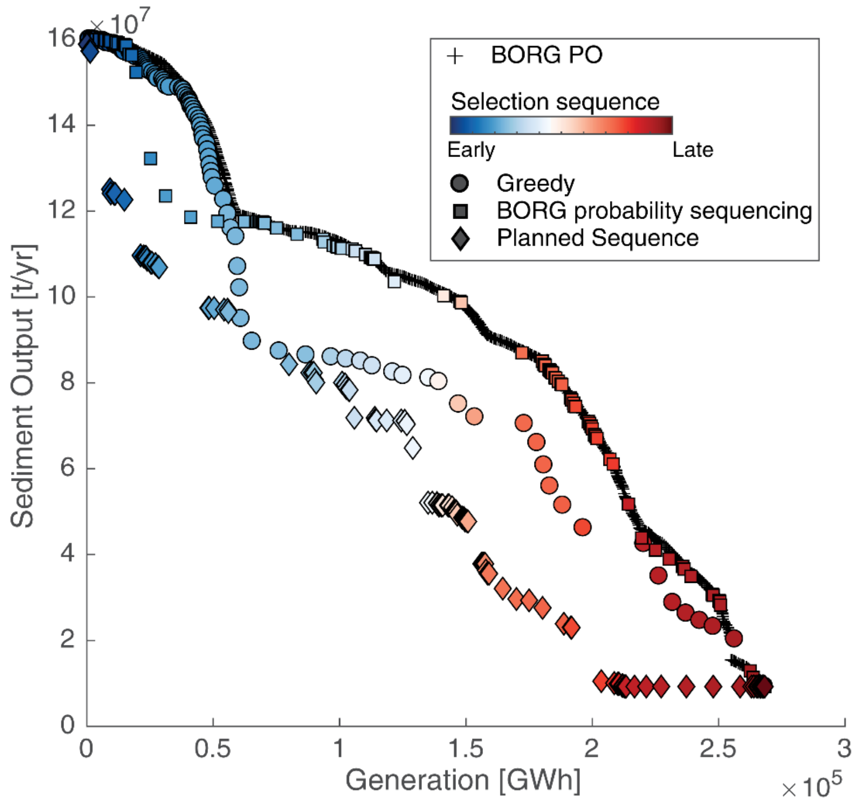

Fig. S9. Comparing BAU and different algorithms for dam sequencing to optimal dam portfolios. Comparing the trade-offs between sediment transport and hydropower generation along the actual and planned dam sequence (diamond markers) with a greedy algorithm (round markers), individual Pareto-optimal (PO) portfolios derived from BORG (PO, crosses) and the dam sequence derived using probability sequencing (PS) based on BORG-derived portfolios (square markers). See fig. S10 for the spatio-temporal layout of dam sequences derived from the Greedy algorithm and the probability sequencing.

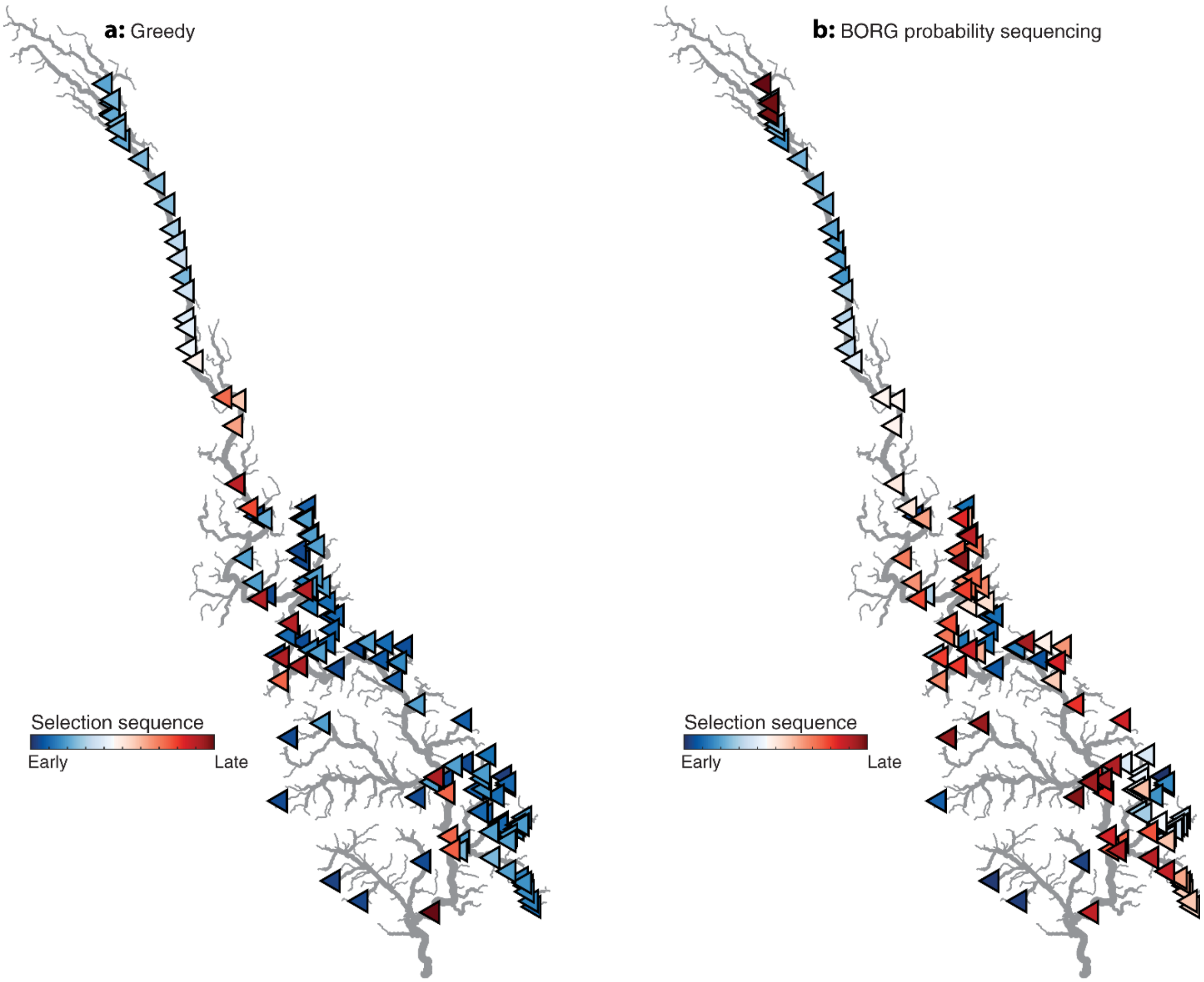

Fig. S10. Spatiotemporal dam sequences from different algorithms. Selection sequence of dams derived using either a greedy algorithm (a) or the BORG probability sequencing (b). The color code indicates when a dam site (triangle markers) is included in the optimal development sequence.

Supplemental Methods

Supplementary Method 1. Building the dam database

This study is based on a comprehensive dam database that is available as data S1. For dams in the lower Mekong countries we used tabulated information on dam sites provided by the Mekong River Commission (*4*). For dams in the Chinese Lancang (Upper Mekong), we referred to spatially referenced dam databases provided by International Rivers and Open Development Mekong (*5*, *37*) and compared the data to dams listed in Räsänen et al. (2017) (*44*). This resulted in a raw database of 199 dam sites.

Geo-referencing

First, we removed 31 dam sites from the database for which no coordinates were available. We also removed dam sites more than five kilometers away from river channels considered in the CASCADE model. Notably, the removed dam sites are very small. For example, in China we removed dams with a combined annual generation of 24 GWh/yr, very little compared to a total of 155000 GWh in China.

Missing inflow data

The resulting dam database remained fragmentary with regard to some key parameters. In a previous paper (*18*), we reported 235000 GWh/yr as generation potential from major dams in the basin, considering only dams that had all information available and that might hence be more likely to be built in the near future. As we had the ambition to explore all hydropower options for the basin, we aimed to complete the dam database as much as possible using various regression and spatial analysis techniques. Specifically, 16 dam sites in China lacked information on gross storage and 28 dams had no information on the inflow, both prerequisites to calculate the trap efficiency.

To estimate the inflow for dam sites, we derived the drainage area at all dam sites from the DEM and fitted a regression between the tabulated inflow and drainage area for these sites. The resulting linear regression resulted in a very good fit between drainage area and inflow (R^2=0.95, fig. S1). We then used that regression to interpolate missing mean annual inflow data.

Missing mean annual generation

For 12 dams, data on mean annual generation were missing. Therefore, we fitted a linear regression between tabulated installed capacity and mean annual generation. The resulting linear regression resulted in a very good fit between installed capacity and mean annual generation (R^2=0.97, fig. S2).

Missing gross storage data

For dams without information on gross storage volume, we approximated the storage from a digital elevation model (DEM) and the tabulated full supply level (FSL), respectively the dam height, if the FSL was not available. We first identify all cells draining towards the dam location by applying a d-8 flow routing algorithm (*40*). Of all these cells, only those with an elevation lower than the FSL are inundated in the reservoir, forming a set of inundated cells, $C_i(d)$, for dam d. For any inundated cell, $c_i \in C_i(d)$, we determine the elevation difference between the FSL and the DEM surface. If $z(c_i)$ is the DEM surface elevation in cell c_i, the impounded volume in cell c_i is defined as

$$V(c_i) = \left(FSL - z(c_i)\right) * A(c_i)$$

Where $A(c_i)$ is the area of a grid cell in the DEM. The total reservoir volume of reservoir R is then defined as sum of $V(c_i)$ over $C_i(d)$

$$V_{tot} = \sum_{c_i \in C_i(d)} V(c_i)$$

Supplementary Method 2. Detailed description of the CASCADE model

Calculating the sediment supply to the Mekong Delta for a dam portfolio P requires, first, information on natural sediment transport in the network and, second, information on the sediment trapping in each dam. Based on this information, we calculate the cumulative trapping in all dams belonging to a portfolio. We represent the natural sediment transport in the Mekong Basin using a combination of the CASCADE framework for sediment connectivity (*23*), combined with estimates for sediment yield from different geomorphic provinces of the Mekong Basin (*8*).

The procedure is as follows: Kondolf et al. (2014) (*8*) divided the Mekong river basin in nine geomorphic provinces based on topography, climate, and lithological parameters. Based on an estimated 160 Mt/yr of total sediment supply to the delta, they assigned a specific sediment yield [t/km^2/yr] to each geomorphic province, such that the total load at the basin outlet matched the estimated 160 Mt/yr. In contrast to Kondolf et al., (2014) (*8*), who did not include an explicit network-scale routing scheme, we here combined estimates of sediment yields with the CASCADE framework for sediment routing. It should also be noted that there is a recent debate around the total sediment load of the basin mostly resulting from scarcity and uncertain calibration of sediment measurements (*45*). However, the results of the analysis presented herein will hold as long as the relative contribution of each geomorphic province estimated by Kondolf et al. (2014) is accurate. With that regard, there is good evidence that sediment from the Lancang and the Tertiary Volcanic Plateau contribute the highest percentage of the basin's load, as assumed in this study (*46–48*).

We first derived the drainage area and the river network from a 250 m resolution DEM (*39*) using standard procedures (*40*). Because the drainage area derived from the DEM is ca. 10 % larger than the manually delineated geomorphic provinces we scaled the values from Kondolf et al. (2014) such that the sediment supply to the Mekong Delta is 160 Mt/yr. We used 150 km^2 as threshold of channel initialization, resulting in a network with a total length of 29,534 km. We split the network at each confluence and at each potential dam site, resulting in 845 separate reaches with an average length of 35 km. We then calculated the direct drainage area of each reach from the DEM. We represent sediment supply from the direct drainage area of each reach as an individual sediment source ς in the CASCADE model. Sediment supply for each source, $\Theta^{\varsigma}_{(S,in)}$, is calculated as

$$\Theta^{\varsigma}_{S,in} = A_D(\varsigma) * y(\varsigma)$$

Where $A_D(\varsigma)$ is the area that drains directly into the reach to which ς is associated, and $y(\varsigma)$ is the sediment yield in the geomorphic province where ς is located (see fig. S3 a for the geomorphic provinces

and the spatial distribution of $\Theta_{S,in}^{\varsigma}$ and fig. S4 a for a definition sketch of the model showing the role of sediment supply, $\Theta_{S,in}^{\varsigma}$).

Second, we use the CASCADE framework to route the sediment from each sediment source through the downstream network and to the basin outlet, denoted Ω, resulting in a spatially distributed estimate of sediment flux in each reach (see fig. S3 b). CASCADE is a spatially-distributed sediment connectivity model that was previously used to calculate sediment connectivity between multiple sources of sediment and the downstream river network based on empirical sediment transport formulas (*23*, *49*). In this application, we reduced CASCADE to its graph-based routing scheme and did neither consider that different sources might supply different grain sizes, nor did we calculate the transport capacity of the network with the current or future hydrology which might change due to climate change and as dams alter the Mekong's flow regime (*15*).

CASCADE conceptualizes the river network as a directed graph. This graph consists of a set of E edges, and N nodes, where an edge e, represents a river reach in the model (i.e., there are 845 edges in the model). Each edge is then assigned a single sediment source ς, with a supply rate $\Theta_{S,in}^{\varsigma}$ (fig. S3 a, fig. S4 a). There are two key measures of sediment flux. First, $\Theta_{S,e}^{\varsigma}$, describes the connected sediment flux between ς and any downstream edge e, or the basin outlet, Ω ($\Theta_{S,\Omega}^{\varsigma}$) (fig. S4 b). Second, each edge can have multiple sediment sources upstream, so that the total sediment flux in an edge, $\Theta_{S,e}$, is the result of sediment flux originating from all upstream sources

$$\Theta_{S,e} = \sum\nolimits_{k \in \Gamma_e} \Theta_{S,e}^{k}$$

Γ_e is the set of all sediment sources located upstream of an edge e, and the above sum hence describes the total sediment flux from all upstream sediment sources to edge e, or from all sources the basin outlet ($\Theta_{S,\Omega}$) (fig. S4 b).

If there are multiple dam sites 1 … i between source ς and a downstream edge e for portfolio P, that portfolio of dams will cumulatively trap sediment and reduce from ς to e, or the basin outlet Ω as a function of the trap efficiency (TE) of the dams (see Material and Methods, and fig. S4 c, e.g., Cascades 3 and 4).

Supplementary Method 3. Calculating trap efficiencies

The trap efficiency at each dam site, $TE(d)$, can be calculated with various methods. If grain sizes of incoming sediment, geometry of the reservoir impoundment, and the operating policy of the dam are known, process-based hydro-dynamic 1D, 2D, or 3D models can be applied to calculate how much sediment can pass the impoundment and can then be discharged to the downstream river network (if a dam is equipped with bottom outlets). However, in data-scarce environments, where neither the grain size of sediment transported in the river network nor bathymetries and operation rules of built and future reservoirs are known, empirical approaches such as the Brune method are commonly applied (*8*, *14*, *50*)

$$TE_{Brune}(d) = 1 - \frac{0.05}{\sqrt{CI(d)}}$$

with CI being the ratio of mean reservoir storage volume $V(d)$ to total annual inflow $Q(d)$

$$CI(d) = \frac{V(d)}{Q(d)}$$

While there are several other empirical approaches, such as the Brown or Churchill equation, we applied the Brune Method to be consistent with previous studies of dam trap efficiency in the Mekong (*14*, *8*) and worldwide (*42*). It should be noted that this approach does not consider for possible drawdowns of a reservoir or other operational strategies which could reduce sediment trapping in a single dam.

The fraction of sediment passing a reservoir, i.e., the release efficiency (*50*) is then defined as

$$RE(D) = (1 - TE(D))$$

The Brune method computes the trapping of fine sediment that is transported in suspension, only. The Mekong, instead, supplies a relevant amount of coarser sediment, i.e., sand and even fine gravel, to its delta (*51*). This sand, of utmost importance to build the coast and the morphologic structure of the delta (*7*), will mostly be trapped, especially in larger dams (*52*). Hence, we modified the calculation of the Brune trap efficiency to include also sand and coarser bedload. I.e.,

$$TE_{BM} = 1$$

Where TE_{BM} is the trap efficiency for bed material. We also assume that the average fraction of bed-material load versus total load, f_{BM}, in the Mekong is 10 % of the total load, in accordance with local observations and typical global values (*43*, *47*). The total trap efficiency of a dam becomes

$$TE(d) = \left(\underbrace{1 - f_{BM}}_{Suspedend\ load} \right) * TE_{Brune} + f_{BM} * TE_{BM}$$

i.e., a sum of trap efficiency weighted for the fractioning of total load between suspended and bedload. It should be noted that f_{BM} likely varies throughout the basin. This spatial variability can be included in the future if more detailed data on sediment transport would be collected.

Supplementary Method 4. Quantifying performance of past and future dam sequences

To represent the planned sequence of dams, we first grouped all dam sites according to their current status (built, under construction, planned) and sorted each group by their COD year. If no COD date could be identified, we set the COD date to 2030. We then sorted each group according to the dams' commercial operation date (COD) (fig. S5 a). The addition of each dam results in an updated portfolio (roman numerals in fig. S5 b). We then simulate the performance of the dam portfolio following that sequence, i.e., after addition of each new dam, to derive a trajectory of performance with regard to objectives J_1 and J_2 (fig. S5 b). The dam portfolio resulting after the addition of a dam can be assigned a year, based on the COD date of that last dam added (fig. S5 b).

Supplementary Method 5. Transferring optimal dam portfolios into optimal dam sequences

This section proposes a straightforward way for strategic, "no-regret", dam sequencing. We use the term "no-regret" in the sense that dams are sequenced such that the portfolio resulting after the addition of each dam creates a trade-off that is as close to a Pareto optimal portfolio as possible.

This approach for identifying dam sequences is also beneficial if a certain dam portfolio was selected for development, as demonstrated in fig. S6 b, c, and d. For example, assume that the projected energy demand is equal to the generation of portfolio C in fig. S6 b. Being Pareto optimal, Portfolio C provides an optimal trade-off between generation and environmental impact. It includes three dam sites, 2, 4 and 5. These dam sites can be developed in multiple different sequences (fig. S6 c). These sequences are equivalent in terms of the final portfolio, the final energy generation and the final environmental impact, resulting in the trade-off marked in fig. S6 a, Portfolio C. However, the sequences are not equivalent in terms of intermediate trade-offs. For example, Sequence 3 would first develop the most downstream site included in Portfolio C (site 2) (fig. S6 c) and then more upstream sites 5 and 4. This would lead to an early increase in generation and also a large drop in environmental performance (fig. S6 d, dashed line with grey diamond markers). Developing sites 4 and 5 (sequence 3) before site 3 would lead to less environmental impacts for low and intermediate generation (fig. S6 d, solid black line with square markers). As mentioned above, any sequence will result in the same dam portfolio and hence in the same trade-offs if all dams are built (fig. S6 d, small network). We argue, however, that sequence 2 is more adaptive and hence preferable as it dominates sequence 3 for intermediate levels of generation. For example, if the demand growth is insufficient to justify the development of all three sites and development is stopped earlier, the combination of sites 4 and 5 will result in less impacts than the early development of site 2.

For the Mekong Basin, for example, the final, highest-generation portfolio of all sequences is identical – if all dams are built the sediment trapping and generation does not differ between the final dam portfolio and the sequence does not matter. However, as discussed in the paper, an optimal sequence leads to better trade-offs for intermediate levels of hydropower generation.

To conclude, dam sequencing has two benefits.

1) Find dam portfolios that form a sequence of increasing energy generation, while minimizing environmental impacts.
2) Determine the sequence with which to build the dams within a certain dam portfolio.

Dam sequencing using a multi-objective evolutionary algorithm

The proposed approach is based on a post-processing of PO portfolios derived using the BORG multi-objective evolutionary algorithm (*24*), and based on the assumption that dams occurring in many portfolios should be built earlier than dams that are included in fewer portfolios. This approach can be used with dam portfolios derived using any kind of optimization approach.

The need for and benefits of this spatio-temporal sequencing are shown in fig. S7 a, which shows Pareto-optimal dam portfolios (identified by capital letters) with different trade-offs between an environmental and an energy objective. Each of these portfolios consists of a different spatial configuration of dam sites (fig. S7 b). For example, portfolio D in fig. S7 b does not include all dam sites from portfolio C. Hence, portfolios do not necessarily form a practical sequence to increase energy generation.

To derive an optimal dam sequence, we first calculate with which probability $\mathcal{P}_d$ a dam site d occurs in a PO portfolio, i.e., $\mathcal{P}_d(d \in P_{PO})$ (fig. S7 c) Second, we sort all dam sites according to $\mathcal{P}_d$. This results in a rank $\mathcal{R}_d$ of each dam site d in the development sequence so that $\mathcal{R}_d = 1$ is, for example, the first ranking site. We then arrange the dams such that

$$\mathcal{R}_d = 1, \mathcal{R}_d = 2, \dots, \mathcal{R}_d = N_{Sites} - 1, \mathcal{R}_d = N_{Sites}$$

so that the order of subscripts d would identify the optimal dam sequence (fig. S7 d). We assign an equivalent COD year to each dam in the sequence by comparing the hydropower generation for each step in that sequence to the hydropower generation along the past and planned sequence.

Finally, we compare tradeoffs along different sequences against the PO portfolios derived using BORG. We also evaluated how Pareto sequencing performs compared to a simple greedy algorithm (Supplemental Results), which would not require a previous optimization.

fig. S7 c - e show the extraction of the optimal development sequence from the Pareto-optimal portfolios (fig. S7 a and b). Each portfolio can be represented as a row in the decision matrix (fig. S7 c), a value of 1 indicates that a dam site is included in the portfolio (compare fig. S7 b with c). Based on that matrix, we determine the probability that a dam site is included in any portfolio. We propose that dams that are included in most of the portfolios should be built first, and dams that are included in least portfolios should be built last, creating a sequence of development (fig. S7 d). If dams are included in the same number of portfolios, we consider the site with the higher generation first. Finally, we re-simulate the performance of the development sequence (fig. S7 d), resulting in a new trade-off curve (fig. S7 e). This approach is hereafter referred to as BORG probability sequencing, or BORG PS.

Dam sequencing using a greedy algorithm

The greedy algorithm performs a stepwise evaluation of sediment trapping. In each step, the algorithm separately adds each remaining dam sites to the portfolio of dam sites selected in the previous step. For example, in the first step (fig. S8 a) the performance of all dams is evaluated. The dam site with the lowest impact is selected (site 4). In the step 2, the performance of all portfolios resulting from combining site 4 with all remaining sites is evaluated (fig. S8 b). This process is repeated until all dam

sites are selected. The dam sequence is then determined as the order in which the dams have been selected (fig. S8 c).

Formally, let s be the counter for the steps. Then, the algorithm minimizes the problem of the form

$$\min_{u}(-J_2(P(s-1), u))$$

Where J_2 is the objective value for sediment supply to the delta, $P(s-1)$ is the dam portfolio resulting from the previous step, and u is the decision of the current step. u is a binary decision vector. $u \notin P(s-1)$, i.e., u can be any dam site except the sites that were added to the dam portfolio in previous steps.

In pseudo code, the algorithm can be written like

$for\ s = 1 \ldots N_{sites}$ -> Loop through all dam sites

$for\ u \notin P(s-1)$ -> Loop through all dam sites that are not yet included in the present dam portfolio

$-J_2(P(s-1), u)$ -> Evaluate the objective function resulting from adding each of these dam sites to the dam portfolio from the previous step.

$end\ for\ u$

$P(s)$= $\{P(s-1), \min_u(-J_2(P(s-1), u))\}$ -> add dam site that creates a dam portfolio with minimal increase of sediment trapping.

$end\ for\ s$

Supplementary Method 6. Deriving active channel width

Active channel width, $W_{AC,e}$, is required for each edge in the network to estimate the Sediment Reduction Index (SRI). Similar to Schmitt et al. (*23*), we used a regression to determine active channel width as a function of slope and drainage area of a reach

$$W_{AC,e} = {A_{D,e}}^{m} * {I_e}^{n}$$

Where $A_{D,e}$ is the drainage area and I_e is the mean gradient of e. Both, $A_{D,e}$ and I_e can be extracted from the DEM. m and n are regression parameters that are fitted to observations of W_{AC} using a least-square regression. To derive observation samples of W_{AC}, we randomly selected 30 reaches of each

stream order (i.e., from 1 to 6), that is 180 reaches, or 21 % of 845. We measured the active channel width at least once (for smaller reaches of visibly homogenous width) and up to 3 times, when there was a major variability in channel width visible from the satellite imagery or for very long reaches, resulting in a total of 287 samples.

We found a best fit for $m = 0.59$ and $n = -0.08$, which follows the expected positive correlation between active channel width and drainage area and the negative correlation between active channel width and slope. The correlation coefficient of that fit was relatively low (R^2=0.42). Despite the considerable scatter around a line of perfect fit the residuals of the regression were well distributed with a t-test showing that the distribution of residuals is normal and the residual mean is not significantly different from zero at a 0.01 confidence level. As this regression is not used for any hydraulic calculations, we propose that this regression presents a suitable estimate for the basin-wide spatial variability of active channel width (e.g., with higher-order low-land channels being wider than lower-order upland channels), which in the future could be replaced by remotely-sensed data.

Last, the extracted river network does not contain the delta. The delta's complex flow pattern with diverging channels and distributaries does not allow for deriving a river network with flow accumulation algorithms. Hence, we delineated major distributary channels in the delta manually and measured their total length. We derived a value of 600 km. The interpolation of active channel width is impossible in the delta, as neither drainage area (because of diverging flow patterns) nor slope (because of the low relief and potentially high vertical errors in the DEM) can be derived. Hence, we assigned the delta channel network a width equal to the width at the outlet node of the delineated network. These measurements are likely conservative, underestimating the SRI index in Vietnam, given the intricate network of natural and manmade channels in the delta that might be impacted by sediment starvation (*2*).

Supplemental Results

Supplementary Result 1. Performance of different sequencing algorithms

The Pareto-optimal (PO) portfolios identified using the BORG MOEA set the benchmark for testing the sequencing algorithms and the input for the PS algorithm. Even neighboring PO portfolios do not necessarily contain similar dam sites, so that PO portfolios alone cannot guide a development sequence. The performances of the planned development sequence, the PO portfolios, the greedy algorithm sequencing, and the BORG probability sequencing are shown in fig. S9.

Comparing the greedy algorithm to BORG-PS, we find that both algorithms perform differently for reproducing different parts of the Pareto front (fig. S9, cross markers). The greedy algorithm first selects all tributary dams (fig. S10 a), resulting in a sequence that is very close to the Pareto front for a generation of up to around 50,000 GWh/yr (fig. S9, circle markers). Then, after all smaller dam sites have been included in the dam sequence, dams in the Lancang are selected from upstream to downstream, resulting in a Pareto-dominated trade-off (i.e., with worse trade-offs than the Pareto front), not much superior to the historic and planned sequence. For a generation between 60,000 GWh/yr and 200,000, the greedy sequence dominates the planned sequence but is clearly dominated by the Pareto front.

For lower generation (0 – 50,000 GWh/yr), BORG PS derives a sequence dominated by both the Pareto front and the greedy sequencing (fig. S9, square markers). This is because BORG PS selects some dams on the upper and middle Lancang earlier than the greedy sequence (compare colors between fig. S10 a and b). Lower Mekong tributary dams are selected in a more heterogeneous pattern than for the greedy sequence. In general, hydropower cascades along tributaries are selected at a similar time. The greedy algorithm instead selects dams more along an upstream-downstream pattern (more upstream dams in all tributaries are developed earlier than more downstream dams). From a real-world perspective, BORG PS might hence be preferable as dam development is focused on certain tributaries, while other tributaries are left free-flowing. Beyond 50,000 GWh/yr generation, the BORG PS clearly dominates the greedy sequence and trade-offs are nearly identical to individual PO portfolios. We hence used the BORG PS throughout this paper as its performance is superior to the greedy algorithm for ranges of generation most relevant for this research (i.e., starting from the currently installed generation capacity of around 135000 GWh/yr).

Finally, these results provide evidence for that it might not be possible to fully translate the performance of separate PO portfolios (*18*, *20*) into a dam sequence that exactly matches the performance of individual PO portfolios (conceptually shown in fig. S7). Optimal low generation portfolios (less than around 50,000 GWh/yr generation) include mostly tributary dams. Higher generation Pareto-optimal portfolios include instead major dams on the Lancang. From a sequence perspective, this means that following the PO sequence would require to first develop smaller dams and to then remove them to reach an optimal trade-off over the entire Pareto front.

These results also highlight the usefulness to compare different sequencing approaches for a specific case study in order to select the most appropriate approach.
